# Role of Estrogen Receptor β, G-Protein Coupled Estrogen Receptor and Estrogen-Related Receptors in Endometrial and Ovarian Cancer

**DOI:** 10.3390/cancers15102845

**Published:** 2023-05-20

**Authors:** Susanne Schüler-Toprak, Maciej Skrzypczak, Carsten Gründker, Olaf Ortmann, Oliver Treeck

**Affiliations:** 1 Department of Obstetrics and Gynecology, University Medical Center Regensburg, Caritas-Hospital St. Josef, 93053 Regensburg, Germany; olaf.ortmann@ukr.de (O.O.); otreeck@csj.de (O.T.); 2 Second Department of Gynecology, Medical University of Lublin, 20-954 Lublin, Poland; skrzypm@yahoo.co.uk; 3 Department of Gynecology and Obstetrics, University Medical Center Göttingen, 37075 Göttingen, Germany; grundker@med.uni-goettingen.de

**Keywords:** estrogen related receptor, estrogen receptors, G protein-coupled estrogen receptor, ovarian cancer, endometrial cancer

## Abstract

**Simple Summary:**

Despite new therapeutic approaches, ovarian cancer is still the most lethal gynecological cancer that is mainly diagnosed in its advanced stages. Contrarily, endometrial cancer is often detected in its early stages. However, in the cases of recurrence or advanced disease, treatment options are still limited. Both the ovary and endometrium are affected by estrogens and their receptors. The well-known estrogen receptor α (ERα) mediates estrogen effects such as the activation of cell proliferation. In contrast, the functions of the later discovered ERs, ERβ and GPER1, and of estrogen-related receptors (ERRs), are less understood. Increasing evidence suggests them to be involved in tumor development, progression, and metastasis. This article provides a summary and update of the current findings on the role of these receptors in ovarian and endometrial cancer to show at which points further research is reasonable and might change the future of their treatment.

**Abstract:**

Ovarian and endometrial cancers are affected by estrogens and their receptors. It has been long known that in different types of cancers, estrogens activate tumor cell proliferation via estrogen receptor α (ERα). In contrast, the role of ERs discovered later, including ERβ and G-protein-coupled ER (GPER1), in cancer is less well understood, but the current state of knowledge indicates them to have a considerable impact on both cancer development and progression. Moreover, estrogen related receptors (ERRs) have been reported to affect pathobiology of many tumor types. This article provides a summary and update of the current findings on the role of ERβ, GPER1, and ERRs in ovarian and endometrial cancer. For this purpose, original research articles on the role of ERβ, GPER1, and ERRs in ovarian and endometrial cancers listed in the PubMed database have been reviewed.

## 1. Introduction

Estrogens, as the main female sex steroids, have important functions in the regulation of growth, differentiation, and other physiological processes in the human ovary and endometrium [1]. Besides the long-known estrogen receptor (ER) α, the effects of estrogens are also mediated by the later discovered ERβ, another member of the nuclear receptor superfamily. Both estrogen receptors, coded by the genes *ESR1* and *ESR2*, primarily act as ligand-activated transcription factors that directly bind to DNA at specific estrogen response elements (EREs), thus regulating the transcription of their target genes [1]. In addition to this direct genomic action, they also trigger the activation of cytoplasmic kinase cascades, which ultimately also result in gene regulation [2]. The tumor-promoting role of ERα in many estrogen-dependent cancers has been well studied. In contrast, the role of ERβ in cancer is less understood, although several studies suggested this receptor to have tumor-suppressive functions in breast and prostate cancer cells [1]. ERβ expression has been detected in the ovary and uterus, in luminal and myoepithelial cells of the human breast, in subcutaneous adipose tissue [3], and in tissues of the prostate, testis, and brain [4]. Alternations in ERβ expression and signaling have been discussed in the context of physiological and pathological processes [5,6,7,8,9,10,11,12] and were found to be involved in the pathophysiology of various tumors [13,14,15,16,17,18,19,20,21]. In certain situations, ERβ acts as an ERα antagonist. Therefore, their ratio has an influence on their effects [22,23,24]. Moreover, splice variants of the *ESR2* gene (ERβ1, ERβ2, etc.) activate cancer cells in various ways [25,26,27]. We, and others. published several studies on the role of ER in gynecologic malignancies that indicate differential effects of this receptor in ovarian and endometrial cancer [28,29,30,31,32].

In addition to ERα and β, the G protein-coupled estrogen receptor (GPER1) mediates estrogen effects not as transcription factor binding to EREs, but via non-genomic signaling. This seven-transmembrane receptor, formerly known as GPR30, has several mechanisms of action. On the one hand, it mobilizes calcium and initiates cAMP synthesis. On the other hand, it transactivates the epidermal growth factor receptor (EGFR) which induces PI3K and MAPK signaling pathways and other mechanisms [33]. From these mechanisms, GPER1 signaling ultimately leads to gene regulation affecting cell-cycle progression as well as the proliferation, differentiation, apoptosis, migration, and invasion of the cells, making it an important player in carcinogenesis [33].

Estrogen signaling is affected by estrogen-related receptors (ERRs), termed orphan receptors. They are also members of the nuclear-receptor superfamily with three existing subtypes, ERR α, β, and γ. Although they have a strong homology with ERα, they cannot directly bind endogenous estrogens. Therefore, these receptors regulate gene expression via post-translational modification or availability of transcriptional co-regulators rather than ligand binding [34,35]. Moreover, the regulation of their expression alters their mode of action during carcinogenesis [35]. These three subtypes exhibit distinct impacts not only on physiological signaling or on the metabolic disorder type 2 diabetes mellitus, but also on the carcinogenesis of hormone-related cancers [34]. For example, ERRα and ERRγ contribute to the regulation of bioenergetic processes, whereas ERRβ monitors placental development and stem cell maintenance [34]. 

Ovarian cancer (OC) is the most lethal gynecological malignoma. In the majority of cases, it is diagnosed in its advanced stages, as it is characterized by a great heterogeneity, and effective treatment options are sparse. In endometrial cancer (EC), the course of the disease is different [32]. In western countries, this cancer entity is the most commonly reported gynecological cancer, mainly occurring in postmenopausal women [36,37]. Overexposure to estrogens, especially without the addition of progesterone, is considered to be a relevant risk factor [36,37,38,39]. In 1983, Bokhman proposed a dual classification of the EC. Type I EC occurs predominantly in obese women with hyperlipidemia and signs of hyperestrogenism [40]. It is characterized by moderately to highly differentiated tumors that often only infiltrate the myometrium superficially, are highly sensitive to progesterone, and are associated with a good prognosis [40]. Having postmenopausal bleeding as an early symptom enables diagnoses to be made often during the early stages of this condition. In contrast, type II EC is not associated with endocrine or metabolic dysregulation. These are poorly differentiated tumors with a tendency for deep myometrial infiltration, with a high likelihood of pelvic lymph node metastases [40]. Sensitivity to progesterone was 42.5% compared with 80.2% for type I EC in Bokhman’s prospective study of 366 EC patients, respectively [40]. Prognosis was significantly worse with a five-year survival of 58.8% compared with type I EC (85.6%) [40]. In 2013, a new classification of EC was suggested that takes into account molecular characteristics [41]. According to this categorization, EC can be subdivided in POLE ultramutated, microsatellite instability hypermutated, copy-number low, and copy-number high EC [41]. These subtypes have found their way into the fifth edition of the WHO classification of female genitalia subdividing POLE-mutant, MMR deficient, P53 abnormal ECs, and those of no specific molecular profile [42]. ECs with a non-specific molecular profile are often associated with hyperestrogenism such as the classical former type I EC. 

For a long time, ERα was considered to be the main player in estrogen signaling. Today we know that the signaling mechanisms mediating the cellular response to this hormone are much more complex. The following article provides an update on the role of estrogen signaling in the pathophysiology of OC and EC, focusing on ERβ, GPER1, and ERRs.

## 2. Estrogen Receptor β (ERβ)

### 2.1. ERβ in Ovarian Cancer

#### 2.1.1. ERβ Protein and mRNA Expression in Ovarian Cancer and Their Influence on Patients’ Survival

After the initial controversies mainly based on immunohistochemical (IHC) studies using unspecific antibodies, the tumor-suppressive role of ERβ in OC became more and more obvious. 

ERβ expression was found to decline in primary ovarian epithelial tumors compared to normal ovarian tissue as it has been shown in breast, colon, and prostate cancer [43,44,45,46,47,48,49,50,51,52,53]. In their IHC-based study, Lindgren et al. analyzed 53 benign, borderline, and malignant ovarian tumors of different types and found a significantly lower ERβ expression in ovarian cancer tissue compared to normal ovaries [54]. Moreover, the level of ERβ protein expression in ovarian cancers has an impact on the survival of the patients. In the IHC-based study published by Halon et al., a higher ERβ expression (>30% of cells) was associated with increased overall survival time and progression-free time (*p* = 0.00161 and *p* = 0.03255, respectively) compared to patients with lower ERβ expression [55].

Numerous studies determining ERβ expression in OC at the mRNA level via RT-qPCR corroborated the results of IHC studies showing downregulation of this receptor in OC tissue and its association with longer survival. Chan et al. reported a significantly lower expression of ERβ mRNA in 161 OC samples compared to normal ovarian tissues (*n* = 58) and tissues of borderline tumors of the ovary (*n* = 25) [49]. Similar results were obtained in a study by Suzuki et al. when comparing the expression of ERβ in 64 OC specimens with normal ovarian tissue via RT-qPCR [52]. Median expression levels of ERβ were lower in OC tissues than in normal ovarian tissues (*p* < 0.001). In another RT-qPCR based analysis, ERβ expression was found to be significantly lower in tumors of stages II-IV than in those staged I (*p* < 0.001) [49]. OC patients with a higher ERβ expression had a significantly longer disease-free survival (*p* = 0.007) as well as overall survival (*p* = 0.011) [49].

The two ERs, ERα and ERβ, are characterized by a tightly balanced interaction on several levels. ERβ affects the transcriptional activity of ERα. Moreover, both receptors form heterodimers, and bind to EREs present in endogenous hormone-regulated genes among others with different patterns of affinities [56]. Accordingly, small changes in the expression ratio of the two ERs have massive effects on cellular regulatory mechanisms [22,57]. In breast carcinoma, ERα expression is routinely determined to evaluate the possibility of anti-hormonal therapy. This is not yet the case in OC, although strong ERα expression was found to be present in up to 70% of cases depending on the histological subtype [58]. Silvia et al. found low ERα expression in benign ovarian tissue and higher expression in OC specimens in their IHC-based study [59]. However, the fact that they included only 33 OC cases, and 17 benign ovarian samples, makes it impossible to draw definitive conclusions. Another study, but also with low case numbers, showed a similar increase in ERα expression in OC tissue compared to benign ovaries [60]. At the mRNA level, Pujol et al. also observed an increase in ERα expression in OCs compared to benign ovarian tissue [22]. The impact of ERα expression on the survival of OC patients has been discussed controversially. In the large IHC-based consortial study published by Sieh et al. in 2013, they observed that patients with OC of the endometrioid subtype survived significantly longer when their tumors expressed ERα [58]. For high-grade serous OCs, they did not show an association of ERα expression with survival [58]. Bogush et al. performed a quantitative immunofluorescence assay, and found that high expression levels of both ERα (≥25%) and ERβ (≥44%) in the OC samples predicted a signi-ficantly longer progression-free survival (*p* < 0.01) in patients after the first-line treatment of platinum and taxane-based adjuvant chemotherapy [57]. At the mRNA level, Pujol et al. observed an increase in the ERα/ERβ mRNA ratio in OC as compared with normal ovaries and cysts, which mainly resulted from ERβ downregulation [22]. Similar data were obtained by Li et al. at the mRNA and protein levels [23,60]. 

#### 2.1.2. Influence of the Subcellular Localization of ERβ on Its Action in Ovarian Cancer

Cytoplasmic ERs act through non-genomic signaling that interacts with growth factor receptors or cytoplasmic kinases. Therefore, the subcellular localization of ERβ affects its action in OC [50,61,62,63]. An IHC-based study described a shift from nuclear to increased cytoplasmic expression of ERβ during the transition of normal ovarian tissue to OCs when comparing 58 advanced OC specimen with 12 normal ovaries [50]. Our group investigated specimens of 171 OC patients via tissue microarrays. We detected nuclear ERβ in 47.31% of the OC tissues and cytoplasmic expression of this receptor in 23.08%. Compared to better differentiated cancers, nuclear ERβ expression was found to be significantly lower in the G3 subgroup (*p* < 0.01) [62]. Patients with tumors expressing cytoplasmic ERβ survived significantly longer than those with ERβ-negative OCs (chi-square statistic of the log-rank, *p* < 0.05). In a big nested IHC-based case-control study within the prospective Nurses’ Health Study cohorts, of the included 245 OC specimens, 43% showed positive staining for cytoplasmic ERβ, and 71% for nuclear ERβ, respectively [63]. The authors pointed out an inverse association between parity and nuclear ERβ expression (OR, parous vs. nulliparous: 0.46; 95% confidence interval (CI) 0.26–0.81), but not in tumors without nuclear expression of ERβ (OR, parous vs. nulliparous: 1.51; 95% CI 0.45–5.04; *p*_heterogeneity_ = 0.04). Conversely, parity was inversely associated with tumors without cytoplasmic expression of ERβ (OR, parous vs. nulliparous: 0.42; 95% CI 0.23–0.78), but was not associated with cytoplasmic ERβ-positive tumors (OR, parous vs. nulliparous: 1.08; 95% CI 0.45–2.63; *p*_heterogeneity_ = 0.05) [63]. Overall, further investigation of the differential roles of nuclear and cytoplasmic ERβs in OC is needed, as previous controversial findings on the role of ERβ in this malignancy may be, in part, driven by the differential function of ERβ depending on its cellular location.

#### 2.1.3. ERβ Splice Variants and Their Distinct Actions in Ovarian Cancer

The subcellular location of ERβ is not the only factor affecting OC pathophysiology. ERβ mRNA splice variants, partially coding for receptor proteins with an altered function, have been reported to exert different effects on the development and progression of different types of cancer [52,64,65,66,67,68]. Ciucci et al. observed in their IHC-based study, that the cytoplasmic expression of splice variant ERβ2 was found to be associated with a reduced overall survival of OC patients (*p* = 0.006 at the multivariate analysis) [65]. The five-year survival rate was nearly 28% for patients with tumors that expressed cytoplasmic ERβ2, and 60% for patients with cancers without cytoplasmic ERβ2 expression [65]. These data are in line with a more recent study published in 2022. Oturkar et al. performed a tissue microarray with 44 high-grade serous OC specimens and examined the nuclear and cytoplasmic expressions of ERβ2 and p53 [64]. Primary tumors had lower cytoplasmic ERβ2 expression than their metastasis [64]. OC patients with high levels of ERβ2 expression in primary tumors had shorter progression-free and overall survival [64]. All these findings suggest a tumor-promoting, oncogenic role of ERβ2 in high-grade serous OCs. Another IHC-based study analyzed 106 ovarian cancer specimens, and identified ERβ5 as a prognostic marker in ovarian cancer [66]. They found significantly higher nuclear ERβ5 expression in advanced cancers [66]. Higher nuclear ERβ5 and lower cytoplasmic ERβ5 expression were found to be associated with serous and clear cell subtypes, and poor disease-free and overall survival. In contrast to nuclear ERβ5, they demonstrated cytoplasmic ERβ5 as a favorable prognostic marker in ovarian cancer [66]. ERβ5 also attracted attention in studies investigating its expression at the mRNA level. In a study published by Suzuki et al., expression of ERβ subtypes was compared via RT-qPCR in twelve OC cell lines with six primary cultures from ovarian surface epithelium [52]. The authors found a significantly lower expression of ERβ1, ERβ2, and ERβ4 (but not of ERβ5) in OC cell lines [52]. When investigating the mRNA expression in 17 clear cell OC tissues, ERβ5 expression was significantly higher than in those of other histological subtypes, and was found to be comparable to the normal ovarian tissues [52]. Splice variant ERβ5 has also been reported to exert tumor-promoting effects in breast cancer and EC, where its mRNA levels were found to be significantly higher than in normal tissues [32,67,68]. However, considering the small number of investigated OC specimens, it is too early to draw final conclusions on the exact role of ERβ5 in OC. These data point out that once more, further studies are necessary, with higher numbers of included cases that examine the role of ERβ splice variants in serous OC and in other histological subtypes of this tumor entity.

#### 2.1.4. In Vitro Studies on ERβ Action in Ovarian Cancer

Several in vitro studies suggested an ERα-independent tumor-suppressive role of ERβ in OC as it has been described for breast and prostate cancer [32,69,70] (Table 1). In a work from our group, we observed a decrease in growth and motility, as well as an increase of apoptosis of ERα-independent SK-OV-3 OC cells that were stably overexpressing ERβ1. [29]. These tumor-suppressive effects occurred independently from functional ERα as well as of estrogens and were accompanied by notably increased mRNA levels of growth-inhibitory cyclin-dependent kinase inhibitor p21(WAF1) and a considerable reduction of cyclin A2 mRNA levels. Moreover, we observed an upregulation of fibulin 1c, an extracellular matrix protein, which is overexpressed in OC and breast cancer, and involved in the regulation of cellular motility [29,71]. Furthermore, we demonstrated antiproliferative and pro-apoptotic effects of heterologously expressed ERβ in ERα-negative simian kidney COS-1 cells even in the absence of E2 [72]. In line with this, a further study observed ERβ to exert antiproliferative effects on ERα-negative HeLa cells accompanied by a down-regulation of cyclin D1 expression [73], an important cell cycle regulator known to mediate the proliferative estrogen effects by ERα-induced transcriptional activation and to be overexpressed in epithelial OCs [73,74,75,76]. 

Additionally, activation of ERβ in OC cells by treatment with specific ERβ agonists was able to evoke these tumor-suppressive effects in a similar way [15]. OVCAR-3 and OAW-42 OC cells were treated with the ERβ agonists ERB-041, WAY200070, liquiritigenin, and 3β-Adiol, resulting in a significant growth inhibition by up to 31.2%. In contrast, knockdown of this receptor exerted notable growth-promoting effects. Transcriptome analyzes revealed that the ERβ agonists triggered the downregulation of the cancer-related genes *PTCH2*, *ND6*, and *LCN1* [15]. In a recently published study, another newly developed ERβ agonist, OSU-ERb-12 was employed [77]. The activation of ERβ with this agonist impeded OC cell expansion and tumor growth [77].

Numerous studies have shown that cancer stem cells (CSC) not only have an influence on remission, but also on the progression of tumor diseases [78]. Similar to other stem cells, CSCs are able to differentiate and proliferate. Moreover, they seem to be resistant to common cytotoxic therapies [78]. Signaling pathways, cell surface molecules, and various other molecular targets affect the behavior of CSCs. Furthermore, the microenvironment of the tumor, microRNA, differentiation or resistance markers also influence their properties [78]. This makes CSCs relevant therapeutic targets to prevent both recurrence and resistance to initiated therapies [78,79,80,81,82,83]. Banerjee et al. observed that the ERβ agonist OSU-ERb-12 reduced the CSC population in OCs by compromising the conversion of non-CSC to CSC [77]. On molecular levels, they observed a decrease in Snail expression by use of OSU-ERb12. This resulted in an inhibition of the epithelial-to-mesenchymal transition (EMT), which is involved in the formation of new CSCs [77].

Another ERβ agonist, LY500307, was recently investigated concerning its effect on the reduction of OC stemness [79]. In this work, the authors demonstrated not only a potent reduction of cell viability, but also sphere formation, and the self-renewal of OC cells after treatment with LY500307. They also found expression of the tumor suppressor genes *CDKN1A* and *FDXR* to be increased, whereas expression of the stemness markers decreased [79]. Moreover, the tumor initiation capacity of OC cells was reduced after treatment with LY500307 in orthotopic OC xenograft models [79].

In the future, it should therefore be investigated whether these effects on the CSC population are also evoked by other ERβ agonists. Taken together, these current findings suggest that ERβ agonists may be promising therapeutic options in this lethal disease, as they not only decrease tumor progression, but also decrease the risk of relapse and metastasis by affecting CSCs.

Genetic variations could alter the risk of ovarian cancer via changes in estrogen biosynthesis or signaling cascades. Our group investigated the influence of single nucleotide polymorphisms (SNPs) in the promoter region of the *ESR2* gene encoding ERβ on ovarian cancer risk in 184 ovarian cancer samples [84]. Although an influence on disease risk was not evident, the SNP rs3020449 was deemed to be able to influence disease progression [84].

In conclusion, there is increasing evidence indicating ERβ acts as a tumor suppressor in OC (Table 1). Expression of this receptor was demonstrated to be lower in OC tissue than in the normal ovary at both the mRNA and protein levels. Elevated levels of ERβ were reported to be associated with an improved survival. In vitro studies on OC cell lines demonstrated that the overexpression or activation of ERβ by specific agonists led to reduced proliferation, motility, and migration, as well as increased rates of apoptosis of the OC cells, and several studies suggested that these effects were independent from the presence of estrogens and ERα. Several ERβ agonists were also shown to limit CSC and to reduce the risk of metastasis and relapse using this quality. In future studies, the role of the subcellular localization of ERβ must be further elucidated, as well as the function of the different splice variants in OC. Regarding the different OC subtypes, further studies with larger sample numbers are needed to better understand the role of ERβ in these subgroups.

**Table 1:** In vitro studies on ERβ action in OC cell lines (↑ increase; ↓ decrease; KD, knockdown; EMT, epithelial-mesenchymal-transition; and OCSC, ovarian cancer stem cells).

Cell Lines	Experimental Strategy and Results	Suggested Function of ERβ in OC	Reference
SK-OV-3	Overexpression of ERβ1 **growth** ↓ **motility** ↓ p21 mRNA levels ↑ cyclin A2 mRNA levels ↓ fibulin 1c mRNA levels ↑	tumor-suppressive	[29]
OVCAR-3 and OAW-42	ERβ-KD **growth** ↑ Treatment with ERβ-agonists ERB-041, WAY200070, liquiritigenin, and 3β-Adiol **growth** ↓ *PTCH2*, *ND6*, and *LCN1* ↓	tumor-suppressive	[15]
Kuramochi, OVCAR4, OVCAR3, PEO1, and OV2008	Treatment with ERβ agonist OSU-ERb-12 cell **expansion** ↓ tumor **growth** ↓ cancer **stem cell population** ↓ Snail ↓ EMT ↓	tumor-suppressive	[77]
OCSCs from ES2, OV90, SKOV3, OVSAHO, and A2780 cells	Treatment with ERβ agonist LY500307 cell **viability** ↓ **sphere formation** ↓ **self-renewal** ↓ *CDKN1A* and *FDXR* ↑ stemness markers SOX2, Oct4, and Nanog ↓	tumor-suppressive	[79]

### 2.2. ERβ in Endometrial Cancer

#### 2.2.1. ERβ Protein and mRNA Expression in Endometrial Cancer and Their Influence on Patients’ Survival

As mentioned, there is a considerable amount of evidence clearly suggesting ERβ to function as a tumor suppressor in breast cancer and OC. However, in EC, the role of ERβ remains controversial, particularly judged from the results of the IHC-based studies. As mentioned above, several studies which examined ERβ protein levels in the EC tissue and control tissue by IHC are now known to have used antibodies that were not specific for ERβ, but were also to detect ERα. Regarding tissue studies on the mRNA level, conflicting results can emerge from inadequate PCR primer design. In contrast, various in vitro studies employing EC cell lines came to conclusions which were far less conflictive.

To date, several studies exist based on the examination of ERβ mRNA expression in EC tissue by the means of RT-qPCR. A study examining ERβ mRNA levels in benign endometrial polyps reported the mRNA levels of this receptor to be similar to the adjacent normal endometrium [85]. Saegusa et al. found similar ERβ mRNA expression levels when comparing normal (*n* = 40), well-differentiated, and low-differentiated ECs (*n* = 48) [86]. In contrast, in a study by Smuc et al. on 16 EC samples, ERβ mRNA levels in EC tissue were found to be lower compared to adjacent normal endometrium [38]. In line with this, a recent study published by Hojnik et al. found significantly decreased ERβ mRNA levels in EC tissue compared to adjacent normal endometrium when investigating 44 paired tissue samples [87]. Thus, mRNA-based studies reported either unchanged or decreased ERβ transcript levels in EC. However, due to the small number of investigated samples, definite conclusions cannot be drawn [38]. 

At the protein level, Hu et al. examined the expression of ERα and ERβ by IHC in para-tumor eutopic endometrium (*n* = 30), endometrial atypical hyperplasia (*n* = 30), and EC (*n* = 65) with IHC [88]. According to their data, the expression of ERα was higher in atypical endometrial hyperplasia and early-stage EC compared to para-tumor eutopic endometrium. In contrast, the expression of ERβ decreased from para-tumor eutopic endometrium to EC [88], accompanied with an increase in Cyclin D1 and a decrease in p21/WAF1. Thus, the authors concluded that the reduced expression of ERβ during the dedifferentiation process related to the development of EC may suggest a tumor-suppressive function of this receptor by antagonizing proliferative ERα action. Differing results were obtained in another recent study by Hojnik et al. [87]. They evaluated the mRNA and protein expression of ERα, ERβ, and GPER1 in 44 EC samples and adjacent endometrial control tissue using qPCR, Western blot, and IHC [87]. Compared to healthy endometrium, ERα and ERβ mRNA and protein expression was lower in EC tissue [87]. Moreover, they observed a correlation of ERα and ERβ mRNA and protein expression with those of GPER1 [87]. However, due to the small number of included specimens, while these data provided possible indications, definitive conclusions cannot be drawn. Moreover, this assessment differs from the conclusions of an IHC-based study published by Obata et al. [89]. They evaluated the expression profiles of p53 and ERβ of 154 EC patients, and found an independent association of both parameters with metastasis and recurrence using multivariate analyzes [89]. Moreover, patients with tumors highly expressing both p53 and ERβ had significantly shorter disease-free survival compared to women p53-ERβ-negative OCs (*p* < 0.01) [89]. 

#### 2.2.2. ERβ Splice Variants and Their Actions in Endometrial Cancer

The expression of different ERβ mRNA splice variants, partially translated into receptor proteins with altered function, increases the complexity of ERβ signaling and its role in EC. In a RT-PCR-based study by Chakravarty et al., ERβ2 transcript levels were found to be lower in 26 ECs than in 57 samples of proliferative endometrium [90]. In contrast, several groups, including ours, found the expression of ERβ1 and ERβ2 to be unchanged in EC when compared to postmenopausal endometrium [28,91]. In our study, we analyzed 46 EC specimens and 28 normal endometrial tissues using RT-qPCR and investigated the expression of 18 ERβ splice variants, and showed that ERβ5 and three exon-deleted ERβ variants were overexpressed in EC [28]. Overexpression of ERβ5 has been previously reported in EC, but also in breast and OC [52,91,92]. These findings suggested a tumor-promoting role of ERβ5 in ECs, which is supported by the fact that the expression of ERβ5 and of two other ERβ variants was particularly elevated in G3 tumors when compared to G1 or G2 tumors or to postmenopausal endometrium [28]. An oncogenic role of ERβ5 in EC was also suggested by Collins et al. [93]. They observed ERβ5 forming heterodimers with ERα in Ishikawa EC cells, increasing their sensitivity to E2 as a result. The authors speculated that the expression of ERβ5 in endometrial epithelial cells may increase the risk of malignant transformation [93]. Corroborating the suggested tumor-promoting role of ERβ5 in EC, in our study mentioned above, we found ERβ5 to be not only be overexpressed in EC, but also be positively associated with the expression of oncogene MYBL2 [28]. On one hand, our group identified a unique ERβ splice variant with a deleted exon 4, ERβ∆4, which was significantly downregulated in G2 and G3 tumors, and in total EC samples [28]. On the other hand, we found a positive association of seven ERβ splice variants, including ERβ1 and ERβ2, with the expression of oncogene HER2 in EC tissue [28]. In addition, the siRNA-triggered knockdown of total ERβ expression led to a significant decline of MYBL2 mRNA and protein levels in the EC cells. 

Taken together, regarding the role of ERβ variants, several consistent studies suggest that the ERβ5 splice variant exerts tumor-promoting effects in EC. Conflicting results from mRNA-based studies on the role of ERβ1 and β2 in EC might result from an inadequate primer design. Thus, further studies are needed to clarify their potential tumor-promoting role in EC through analyzing their mRNA expression in tissues, or using isoform-specific overexpression or knockout in EC cell lines [32]. 

#### 2.2.3. Impact of the ERα/ERβ Ratio on Endometrial Carcinogenesis

Given that ERβ can form heterodimers with ERα and is known to function as an ERα antagonist in specific settings, the balanced co-expression of both receptors is a crucial factor in endometrial carcinogenesis. IHC-based studies observed a decrease in the ERα/ERβ ratio during carcinogenesis which was caused by both the down-regulation of ERα and the up-regulation of ERβ [94,95]. A decreased ERα/ERβ ratio was found to be associated with ovarian invasion in a large IHC-based study comprising 214 endometrial carcinoma samples [96]. Other IHC studies revealed a decreased ERα/ERβ ratio to be associated with a shorter disease-free and/or overall survival [97,98]. In one of these studies, the association was found to be significant for the ratio of ERα with ERβ1 and ERβ2, (HR 6.4; 95% CI 1.0–40.6; *p* = 0.04 and HR 9.7; 95% CI 1.1–85.3; *p* = 0.04, respectively) [98]. At the mRNA level, low ERα/ERβ ratios were associated with increased tumorous infiltration of the myometrium and higher rates of lymph node metastasis [99,100]. However, data of the effects of the ERα/ERβ ratios on lymph node invasion are still conflicting [95]. Of note, however, ERβ expression alone did not correlate with clinicopathologic features. [96,101]. As demonstrated in previous studies on breast cancer, another hormone-dependent cancer entity, the function of ERβ in EC is also affected by the level of ERα expression. Thus, the analysis of both ERα and ERβ levels in EC tissue is expected to provide superior prognostic information in comparison to ERα alone [32].

#### 2.2.4. In Vitro Studies on ERβ Action in Endometrial Cancer

In contrast to the conflictive data from studies analyzing EC tissue expression, most in vitro studies employing EC cell lines suggest a tumor-suppressive role of ERβ in this tumor entity.

In the study of Hu et al. mentioned above, knockdown of ERβ in ERα-positive Ishikawa EC cells promoted estrogen-induced cell proliferation via the upregulation of cyclin D1 and downregulation of p21/WAF1 expression [88]. The authors of this study suggested a tumor-suppressive role of ERβ in EC. In a study by our group, we observed that the tumor-suppressive effect of ERβ on EC cells was exerted independently from the presence of ERα as it has been shown in breast cancer previously [16,102,103,104]. The knockdown of ERβ by means of RNAi significantly increased the proliferation of both ERα-negative/ERβ-positive HEC-1A and ERα/β-positive RL95/2 EC cell lines (*p* < 0.05 and *p* < 0.01, respectively) [16]. The underlying molecular mechanisms were examined by transcriptome analyzes, which revealed that the knockdown of ERβ in the ERα-negative cell line led to increased expression of several cancer-related genes, including cell cycle regulator *CCNL1* and tumor-promoting *NMPT*, but to repression of genes associated with differentiation, apoptosis, or growth inhibition. In the ERα-positive cell line, ERβ knockdown led to the upregulation of the ERα coactivators *PNRC2* and *VAV3*, the latter being overexpressed in EC [16]. The observed knockdown effects were confirmed by the application of the ERβ antagonists PHTTP and (R,R)-THC, which led to the increased proliferation of both cell lines. These results highlight that ERβ has a tumor suppressive effect at the endometrium, as it does at other tissues, and should prompt studies that further investigate ERβ as a therapeutic target in EC [16].

Estrogens induce proliferation of the endometrium and function as growth stimulators in the context of EC. As estrogen levels are higher in obese women due to the aromatization of androgens by the enzyme aromatase in the adipose tissue, EC risk is therefore considerably increased in women who are overweight. Together with obesity, the so-called metabolic syndrome, consisting of type 2 diabetes, arterial hypertension, and obesity, became a widespread disease. Treatment of type 2 diabetes and prediabetes with metformin, a guanidine, is commonly used. It has been shown that metformin acts a potent inhibitor of proliferation in the ECC-1 and Ishikawa EC cells lines [105]. Zhang et al. investigated the effects of metformin on cell proliferation and ER expression in EC cell lines that are sensitive to estrogen [106]. The utilization of metformin led to a significant decrease in E2-stimulated cell proliferation through the inhibition of the mTOR signaling pathway, along with a significant inhibition of ERα expression and increase in ERβ expression [106]. These data not only support the previously published data on the growth-inhibitory effect of metformin in EC, they also emphasize the tumor-suppressive role of ERβ in this cancer entity.

In conclusion, regarding the various attempts to elucidate the role of ERβ in EC, most in vitro studies provided consistent results clearly suggesting that ERβ functions as a tumor-suppressor in EC (Table 2). In contrast, the results of studies examining ERβ expression in EC tissues remain controversial [107]. One major problem resulting in conflicting IHC studies is the known use of non-specific ERβ antibodies. Furthermore, in many publications, only small case numbers were included [38,107]. Another problem is the fact that the characteristics of the healthy endometrial tissue used as controls are subjected to changes during the female cycle and depend on the menopausal status, which was not considered in all studies [107,108,109].

Considering all available studies, further efforts are needed to examine to what extent the consistent results from in vitro studies, clearly suggesting ERβ to function as tumor-suppressor in EC, can be verified on the tissue level, e.g., through its association with clinical parameters in large patient cohorts, and by novel mechanistical studies which should use the benefits of novel technologies which have led to current multiomics to clarify the role of this receptor in EC.

**Table 2:** In vitro studies on ERβ action in EC cell lines (↑ increase; ↓ decrease; KD, knockdown).

Cell Lines	Experimental Strategy and Results	Suggested Function of ERβ in EC	Reference
Ishikawa	ERβ-KD **proliferation** ↑ cyclin D1 ↑ p21 ↓	tumor-suppressive	[88]
HEC-1A and RL95/2	ERβ-KD **proliferation** ↑ in HEC-1A cells: ERα-/ERβ+: cell cycle regulator *CCNL1* and tumor-promoter *NMPT* ↑ in RL95/2 cells: ERα+/ERβ+: ERα coactivators *PNRC2* and *VAV3* ↑ Treatment with ERβ antagonists PHTTP and (R,R)-THC **proliferation** ↑	tumor-suppressive	[16]

## 3. G-Protein Coupled Estrogen Receptor (GPER1)

### 3.1. GPER1 in Ovarian Cancer

#### 3.1.1. GPER1 Protein and mRNA Expression in Ovarian Cancer, and Their Prognostic Relevance

Studies examining the expression of GPER1 in OC at the protein level by IHC came to conflicting results. Kolkova et al. analyzed a TMA including 40 ovarian tumors and did not observe a correlation between GPER1 staining and clinical stage, histological grade, or patient survival [110]. In line with this, Fujiwara et al. observed no relation between GPER1 expression levels with survival in an IHC-based analysis of 152 OC specimens [111].

In contrast, several studies suggested a tumor-promoting role of GPER1 in OC [111,112,113]. An IHC-based study investigating tissue from 45 patients with ovarian tumors of low-malignant potential and 89 patients with ovarian cancers showed a higher expression of GPER1 in tumors of higher stage and grade [112]. Moreover, Smith et al. reported a significantly lower five-year survival rate in tumors with a high expression of GPER1 compared to those with low-GPER1 expression (33.3% vs. 72.4%, respectively, *p* = 0.001) [112]. Moreover, co-expression of GPER1 with EGFR was associated with a shorter progression-free survival of OC patients [33,111]. Yan et al. observed an overexpression of GPER1 in OC that was found to be positively correlated with the expression of matrix metalloproteinase 9 (MMP-9), which is often associated with increased invasion [113]. 

However, there are also contrary data suggesting tumor-suppressive functions of GPER1 in ovarian malignancies. Ignatov et al. analyzed GPER1 expression in 35 benign ovarian neoplasms as well as in 35 borderline tumors of the ovary and 124 OCs [114]. Benign tumors and those of a low-malignant potential were found to have significantly higher GPER1 expression levels than investigated OCs [114]. Early stage and well differentiated cancers strongly expressed GPER1, which was found in 83.1% of all malignant tumors [114]. Moreover, they observed significantly longer disease-free survival for patients with GPER1-expressing OCs compared to those with GPER1-negative tumors (*p* = 0.002) [114]. In line with this, OC patients with tumors that had high mRNA levels of GPER1 survived longer (HR = 0.86, *p* = 0.057), and had more lifetime without progression (HR = 0.81, *p* = 0.0035) when open-access mRNA and clinical data by bioinformatical online tools were analyzed [115]. Data recently obtained by the study of Fraungruber et al. also pointed in the same direction. They analyzed 156 OC samples immunohistochemically, and found a significant correlation between the WNT pathway modulator Dickkopf 2 (DKK2) and cytoplasmic GPER1 expression (*p* = 0.001) [116]. High co-expression of Dkk2 and GPER1 was associated with better overall survival in OC patients (*p* = 0.024) [111]. These data suggest a prognostic relevance of both pathways, and indicate that therapeutic interventions targeting both estrogen and Wnt signaling pathways may be successful in OC [116]. 

#### 3.1.2. Influence of the Subcellular Localization of GPER1 on Its Action in Ovarian Cancer

As we already have shown for ERβ, the subcellular localization of GPER1 might also influence its role in the carcinogenesis of OC. Kolkova et al. further analyzed the tissue distribution of GPER1 protein in 37 ovarian tumors [110]. GPER1 is known to be localized in the cell membrane as well as in intracellular membranes [117,118]. In OCs, Kolkova et al. observed predominant GPER1 immunostaining of the malignant epithelial cells. OC stroma only had weak or single cell staining [110]. In normal human endometrial tissue, a comparable staining pattern was described [119,120]. In ECs, GPER1 was evident in the plasma membrane but also in the cytoplasm [110]. Consistent with this, a previous work reported intracellular GPER1 trafficking between the plasma membrane and cytokeratin intermediate filaments [118]. Smith et al. did not describe membrane staining, but showed nuclear along with cytoplasmic staining [110,121]. Moreover, other studies only differentiated between the nuclear and cytoplasmic expression of GPER1 [122,123]. Osaku et al. reported that for patients with high-grade serous OCs (*n* = 38), cytoplasmic GPER1 or nuclear GPER1 was associated with poor progression-free survival (*p* = 0.010 or *p* = 0.013, respectively) [122]. Cytoplasmic GPER1 was an independent prognostic factor for progression-free survival in high-grade serous OC patients (HR = 2.83, 95% CI = 1.03–9.16, *p* = 0.007) [122]. However, in view of the small number of investigated specimens, the results of this study must be treated with great caution. Zhu et al. analyzed tissue samples of 110 OC patients via IHC [123]. In the cohort with nuclear GPER1-expressing tumors, the risk of recurrence was found to be significantly higher. The presence of nuclear GPER1 predicted lower overall and five-year progression-free survival in all patients with OC [123]. Cytoplasmic expression of GPER1 was observed significantly more often in advanced OC, however, it did not predict survival [123]. In conclusion, further studies should follow to elucidate the effect of subcellular localization of GPER1 expression on carcinogenesis in OCs.

#### 3.1.3. In Vitro Studies on GPER1 Action in Ovarian Cancer

The reports from in vitro studies employing OC cell lines on the role of GPER1 in OC also remain inconsistent, which partially can be attributed to the employment of different cell line models, but also to the fact that various studies used only one single cell line, which is commonly considered to provide data of limited significance. 

In a recent in vitro study, GPER1, shown to be expressed in SKOV-3 and OVCAR-3 OC cell lines, was activated by the specific agonist G-1, resulting in increased caspase-dependent apoptosis, as well as decreased proliferation via cell cycle arrest in the G2/M phase. These findings were associated with an increased cyclin B1 and Cdc2 expression, as well as the phosphorylation of histone 3, supporting a tumor-suppressive role of GPER1 in OC [114]. In line with these data, in a study from our group, a significant growth stimulation of OVCAR-3 and OAW-42 OC cells after GPER1 knockdown was observed. In both cell lines, treatment of these cells with the GPER1 agonist G-1 reduced growth dose-dependently [115]. Treatment with G-1 elevated the basal caspase 3/7 activity in both cell lines and induced the protein expression of the cell-cycle inhibitor p21/WAF-1 (*CDKNA1*). Transcriptome analyzes using Affymetrix Gene-Chips corroborated the upregulation of *CDKNA1* expression after G-1 treatment and identified 18 genes that were inversely affected by the knockdown of GPER1 and the application of G-1. In general, transcriptome responses after the use of G-1 was associated with decreased growth, whereas GPER-1 knockdown induced signaling pathways associated with increased mitosis rates and inhibited those associated with apoptosis and interferon signaling [115]. In line with this, another previous study reported G-1 to block tubulin polymerization and thereby interrupt microtubule assembly in IGROV-1 and SKOV-3 OC cells, which was followed by cell cycle arrest in the prophase of mitosis, along with a decrease in proliferation of these cells [124]. One of the underlying mechanisms observed was the increased expression of p21Cip1, a cyclin-dependent kinase inhibitor known to be a target of p53 [124]. Moreover, the authors observed a decreased expression of the anti-apoptotic protein BCL-2 as well as higher cleaved PARP and fodrin levels after G-1 application [124]. Taken together, data from these in vitro studies clearly support a tumor-suppressive role of GPER1 in OC (Table 3).

On the other hand, the results of an in vitro study employing a single cell line only, OVCAR-5, suggested a tumor-promoting character of GPER1 in OC. Yan et al. reported that E2 and the selective GPER1 agonist G-1 increased cell motility and invasiveness, and upregulated the production and proteolytic activity of MMP-9 in ERα-negative/GPER1-positive OVCAR-5 cells [113]. In OVCAR-5 cells, knockdown of GPER1 by means of siRNA and application of the pertussin toxin (PTX), a G-protein inhibitor, led to decreased migration and invasion, as well as reduced MMP-9 expression [113]. Moreover, the same group observed an increased cell number in the S-phase by 17β-estradiol and G-1, resulting in elevated proliferation [125]. Regarding the underlying molecular mechanisms, the authors found that both substances increased the expression of c-fos and cyclin D1. These results are contrary to those published by Albanito et al., who also only employed one cell line. In their study, only E2 or G-1 increased c-fos and extracellular signal-regulated kinase (ERK) expression in ERα-positive BG-1 OC cells when both ERα and GPER1 were available [126]. Moreover, they observed that this induction of c-fos and ERK by either ligand was decreased by the inhibition of the EGFR transduction pathway. This was why they suggested that in these cells, GPER1 signaling relays on ERα expression [126]. Others described that the E2-based activation of GPER1 leads to the transactivation of EGFR and downstream activation of the MAPK and PI3K signaling cascades [127,128]. In OVCAR-5 OC cells, GPER1 knockdown using either siRNA or the G protein inhibitor PTX inhibited basal cell proliferation and attenuated 17β-estradiol- or G-1-induced cell proliferation by a decrease in the S-phase [125]. These findings are supported by other studies showing that GPER1 knockdown by siRNA reduced the cell number to 60% of the siRNA-control-treated cells (*p* < 0.05), while the GPER1 antagonist G-15 inhibited proliferation in two high-grade OC cell lines (KF and UWB1.289) in a dose-dependent manner [122]. Apoptosis of OVCAR-5 cells increased significantly after knockdown of GPER1 with specific siRNA [125]. These findings speak for an oncogenic role of GPER1 in OC cells lacking ERα (Table 3). 

Taken together, the results of these in vitro studies using different cell lines differed strongly, which might result from their ERα status, the presence of E2 in the culture medium, and the fact that the studies using E2 did not address ERβ expression at all. Furthermore, the value of studies using a single cell line only are commonly considered to be limited. However, the conflictive data from these in vitro studies clearly show that further attempts on the cell line level are necessary, preferably employing two or more OC cell lines, to further elucidate the function of GPER1 in OC cell lines. Comparison of the effects of GPER1 and G-1 on the transcriptomes of multiple cell lines will be helpful to discriminate the effects shared between the different OC lines, which may then be able to come closer to the effects seen in OC tissues, from those which are cell-line specific.

**Table 3:** In vitro studies on GPER1 action in OC cell lines (↑ increase; ↓ decrease; KD, knockdown).

Cell Lines	Experimental Strategy and Results	Suggested Function of GPER1 in OC	Reference
OVCAR-3 and OAW-42	GPER1-KD **proliferation ↑** by induction of a mitosis-activating transcriptome **apoptosis ↓** by inhibition of the apoptotic transcriptome Treatment with GPER1 agonist G-1 **proliferation ↓**, by induction of a transcriptome response associated with growth inhibition transcriptome response opposite to GPER1-KD	tumor-suppressive	[115]
SKOV-3 and OVCAR-3	Treatment with GPER1 agonist G-1 **proliferation ↓** by blockade in G2/M phase **apoptosis ↑**	tumor-suppressive	[114]
IGROV-1 and SKOV-3	Treatment with GPER1 agonist G-1 **apoptosis** ↑ anti-apoptotic protein BCL-2 ↓ cleaved PARP ↑ fodrin ↑	tumor-suppressive	[124]
KF and UWB1.289	GPER1-KD or treatment with antagonist G15 **proliferation** ↓	oncogenic	[122]
OVCAR5	Treatment with GPER1 agonist G-1 **cell motility** ↑ **invasiveness** ↑ production and proteolytic activity of MMP-9 ↑	oncogenic	[113]
OVCAR5	Treatment with GPER1 agonist G-1 **proliferation** ↑ protein levels of c-fos ↑ and cyclin D1 ↑	oncogenic	[125]
BG-1	Treatment with GPER1 agonist G-1 **proliferation** ↑ cyclin D1 ↑ cyclin E ↑ cyclin A ↑	oncogenic	[126]

#### 3.1.4. Omega-3 Polyunsaturated Fatty Acids and Shikonin Act via GPER1 in Ovarian Cancer

Several substances that are known to exert tumor-suppressive or -promoting effects in OCs act via GPER1. One study, supporting tumor-suppressive effects of GPER1 in OC was published by Zhao et al. [129]. They investigated the role of omega-3 polyunsaturated fatty acids in OC as tumor-suppressive properties were attributed to them [129]. They observed that eicosapentaenoic acid (EPA) induced the apoptosis of ES2 ovarian clear cell carcinoma cells via GPER1 [129]. As a ligand of GPER1, EPA activated the GPER1-cAMP-protein-kinase A signaling pathway. After knockdown of GPER1 using specific siRNA or its inhibitor G15, the antiproliferative action of EPA was then impaired [129]. Moreover, EPA was able to inhibit AKT and ERK, which led to a decrease in proliferation [129]. In the mouse xenograft model, these mechanisms resulted in a lower tumor volume and weight following the utilization of EPA [129].

Another substance with tumor-suppressive activity is shikonin (SK). SK is one of the major phytochemical components of *Lithospermum erythrorhizon* (Purple Cromwell), which is a type of medicinal herb broadly utilized in traditional Chinese medicine. It is presumed that SK possesses therapeutic actions on various diseases [130]. Liu et al. intended to clarify the underlying mechanisms of SK-promoting apoptosis in OC using gene ontology (GO) and Kyoto encyclopedia of genes and genomes (KEGG) pathway enrichment analyzes [131]. They observed increased apoptotic rates in SKOV-3 and A2780 OC cells after treatment with SK. GO and KEGG analyzes showed that the estrogen signaling pathway was involved in the actions induced by SK [131]. Moreover, treatment of both cell lines with SK decreased GPER1 as well as EGFR, *p*-EGFR, PI3K, and *p*-AKT expression dose-dependently [131]. Use of G-1 increased the pro-apoptotic activity of SK through decreasing EGFR, *p*-EGFR, PI3K, and *p*-AKT expression, whereas these findings were then reversed by use of the specific inhibitor G-15 [131]. The results could also be reproduced in SKOV-3 xenograft models, which showed a G-1 synergistic decrease in tumor growth rates after application of SK that was inhibited by G-15 [131]. Overall, these data suggest GPER1 to be the main target of SK for the induction of apoptosis in OC using the GPER1/EGFR/PI3K/AKT signaling pathway [131].

In summary, GPER1 can exert tumor-promoting as well as tumor-suppressive effects in OC (Table 3). These contrary roles seem to depend on the specific concert of activated and inactivated signaling pathways in tumor cells [132]. Several different signal transduction pathways have been reported to be regulated by GPER1 [132]. Among others, GPER1 activates calcium-signaling, MAP-kinases, PI3-kinase, adenylyl cyclase, Hippo signaling, as well as HOTAIR (HOX transcript antisense intergenic RNA), and inhibits NFκB [132]. Moreover, epigenetic regulation could play a significant role in GPER1 action [133]. Overall, GPER1 is a key player in several signaling pathways that engage in carcinogenesis. Therefore, in the future, the characteristics of these employed cell lines should be considered more closely when investigating the role of GPER1 in OC. 

### 3.2. GPER1 in Endometrial Cancer

#### 3.2.1. GPER1 Protein and mRNA Expression in Endometrial Cancer

As in OC, GPER1 was reported to exert tumor-suppressive as well as oncogenic effects in EC. Our group observed lower GPER1 expression in ECs compared to normal endometrial tissue [134]. We analyzed GPER1 expression and those of nuclear steroid hormone receptors at the mRNA level in 88 EC and normal endometrial tissues [134]. In EC, transcript levels of GPER1 were about 6-fold lower compared to normal endometrial tissue [134]. In line with these data, loss of GPER1 in EC correlated with a higher FIGO stage, higher histological grade, non-endometrioid histology, aneuploidy, lower ERα expression, and disease progression in a study by Krakstad et al. [135]. 

Several parameters influence GPER1 expression, which might lead to inconsistent results. GPER1 mRNA expression varies within the female cycle. In the proliferative phase, higher mRNA GPER1 levels were detected compared to the secretory phase [119]. Moreover, stromal cells had lower GPER1 mRNA expression compared to epithelial cells of the endometrium [119]. Furthermore, E2, 4-Hydroxytamoxifen (OHT), insulin-like growth factor-1 (IGF-1), and insulin are pervasive substances that increase expression of GPER1 [33,136,137,138,139].

Contrary to the results obtained analyzing the mRNA expression of GPER1, IHC investigations suggest a tumor-promoting role of GPER1 in EC. Advanced stage, high grade, or deep myometrial invasion are unfavorable clinicopathological features that were found to have correlated with an elevated expression of GPER1 in EC biopsies that were analyzed via IHC by Smith et al. Moreover, high GPER1 expression was associated with shorter overall survival [33,121]. According to the available data, GPER1 was similarly expressed in type I and II ECs, with no significant differences observed between menopausal status in the luminal and basal surface of the EC epithelium when analyzed by IHC [33,140]. IHC of EC tissues showed that GPER1 expression was greatly increased in endometrial tissues from patients suffering from insulin resistance [137]. Furthermore, GPER1 was expressed at higher levels in patients receiving tamoxifen treatments [33,136].

#### 3.2.2. In Vitro and In Vivo Studies on GPER1 Action in Endometrial Cancer

Controversies can also be observed regarding in vitro results. In a work published by our group, we observed a clear tumor-suppressive action of G-1 in an EC cell line with varying GPER1 expression levels [134]. In GPER1-positive RL-95-2 and HEC-1A EC cell lines, application of G-1 decreased cell growth dose-dependently, whereas no effects were found in GPER1-negative HEC-1B EC cells [134]. 

In other studies, treatment of EC cells with E2 or G-1 exerted oncogenic effects. Du et al. reported for instance, that in Ishikawa and KLE EC cell lines, mRNA and protein expression levels of GPER1 was up-regulated by G-1 and E2 [138]. Down-regulation of GPER1, or the interruption of the MAPK signal pathway, partly or even completely prevented the increase in proliferation induced by G-1 and E2 [138]. 

As we already pointed out, insulin resistance is a frequent finding in EC patients as both diseases are associated with obesity. Lv et al. showed that insulin enhanced estradiol-driven EC cell proliferation by up-regulating GPER1 expression, but not ERα or ERβ. Mechanistically, the authors suggested that insulin upregulated the expression of the DNA hydroxymethylase ten-eleven-translocation 1 (TET1), which increased GPER1 expression via epigenetic modulation [137]. As insulin and insulin-like growth factor-1 (IGF-1) are known to function quite similarly, De Marco et al. observed that in presence of ERα, IGF-1 transactivated the GPER1 promoter sequence and upregulated GPER1 mRNA and protein levels in Ishikawa cells involving the IGF-IR/PKCδ/ERK/c-fos/AP1 signaling pathway [139]. 

Regarding the in vivo data, activation of GPER1 promoted solid tumor formation from RL95-2 cells in a xenograft model of athymic mice [33,141]. Another study set-up observed an inhibition of tumor growth after blockage of GPER1 in an athymic mouse model with a HEC-1A cell line xenograft [33,142]. 

Although there are several controversies concerning the role of GPER1 in EC development, most findings indicate an oncogenic role in ECs (Table 4). A considerable limitation is that in a vast number of studies E2 was used to investigate proliferative effects on EC cells. Thus, a distinction between GPER1-specific effects and reactions that were mediated by other nuclear ERs is not possible. Future studies must address this issue to rule out potential bias. Moreover, in vivo data showing the effects of the inhibition of GPER1 with specific antagonists will be necessary before this promising target will be available as therapy for humans.

**Table 4:** In vitro studies on GPER1 action in EC cell lines (↑ increase; ↓ decrease; KD, knockdown).

Cell Lines	Experimental Strategy and Results	Suggested Function of GPER1 in EC	Reference
RL-95-2, HEC-1A, and HEC-1B	Treatment with GPER1 agonist G-1 **cell growth** ↓ dose-dependently in GPER1 positive RL-95-2 and HEC-1A cells no effect on cell growth in GPER1 negative HEC-1B cells	tumor-suppressive	[134]
Ishikawa and KLE	Treatment with GPER1 agonist G-1 **proliferation** ↑ **invasion** ↑ GPER1-KD or interruption of MAPK signaling pathway **proliferation** ↓ **invasion** ↓	oncogenic	[138]

## 4. Estrogen Related Receptors (ERRs)

### 4.1. ERRs in Ovarian Cancer

#### 4.1.1. ERRα Protein and mRNA Expression in Ovarian Cancer and Its Prognostic Relevance

Knowledge on the role of ERRs in OCs is still sparse. Available studies often include only a few number of OC samples. This is why even published evaluations of ERR expression and its consequences for survival of OC patients is not reliable in all parts. High expression of ERRα was associated with shorter overall survival in a small IHC-based study analyzing 33 OC patients [143]. Another study found ERRα mRNA expression to correlate positively with OC stage [144]. Both data give rise to the impression that ERRα may function as a tumor-promoting factor. Recently, our group established a tissue microarray from 208 OC patients and performed IHC analyzes of OC markers, steroid hormone receptors, and cancer-associated genes [145]. ERRα was detected at different levels in more than 90% of all OC tissues [145]. In our cohort of 208 OC patients, expression of ERRα neither affected overall survival nor progression-free survival [145]. Another group used the cancer public database CPTAC, and showed that in advanced high-grade OC tissues, ERRα expression was higher compared to early low-grade OCs [146]. Moreover, OC cells with a high ERRα expression had greater invasion potential in vitro [146]. Overall, these data suggest a tumor-promoting effect of ERRα in OC.

The master regulator of mitochondrial biogenesis peroxisome proliferator-activated receptor gamma coactivator 1-alpha (PGC-1α) has a major impact on metabolic homeostasis, which in turn is thought to be highly relevant in carcinogenesis [147]. PGC-1α exerts oncogenic effects in some cancer types, as it promotes progression and metastases [147,148,149]. In 42 OC and 31 benign ovarian samples, expression levels of PGC-1α and ERRα were significantly higher in OCs compared to benign specimens (*p* = 0.0059 and *p* = 0.002, respectively) [150]. In OC samples, high expression levels of both ERRα and PGC-1α correlated with both differentiation and lymph node metastasis, as well as CA125 and HE4 levels [150]. Furthermore, women with tumors that were highly expressing both PGC-1α and ERRα tended to have shorter cancer-specific survival (*p* = 0.1276) [150]. However, overall, the number of included cases is too low to draw final conclusions. Also in the study published by Huang et al., the association between ERRα and PGC-1α was investigated [151]. Copy number variations (CNVs) are related to the genetic and phenotypic diversity of cancers [151]. In their recent publication, Huang et al. used data from the cancer genome atlas (TCGA) to explore the associations between ERRα CNVs and histological grade in patients with OC [151]. Data from 620 OC patients were included. They observed a significant association of ERRα CNVs with histological grade (OR 0.6235; 95% CI, 0.3593–0.8877; *p* < 0.05), and PGC-1α CNVs (OR −0.6298; 95% CI −0.9011 to −0.3585; *p* < 0.05) [151]. In multivariate analyses, these associations were found to remain significant [151]. 

#### 4.1.2. In Vitro Studies on ERRα Action in Ovarian Cancer

In vitro studies supported the tumor-promoting role of ERRα in OC. Up-regulation of ERRα using stable vector transfection was recently shown to increase the invasion and motility of the OC cell line HO8010 and its metastatic equivalent HO8910PM, while the use of the ERRα specific antagonist XCT790 weakened these effects [146]. Moreover, ERRα influences EMT in OC. Lam et al. found that the knockdown of ERRα by means of siRNA decreased expression of the EMT-inducer Snail with involvement from the miR-200 family [152]. Finally, inhibition of ERRα with the orthotopical injection of luciferase-labeled ERRα siRNA-expressing SKOV-3 cells decreased tumor mass, ascites, and peritoneal carcinomatosis in vivo [152]. These data suggest that ERRα expression increases the aggressiveness of OCs, making its inhibition a potential therapeutic approach in OC [152].

#### 4.1.3. Role of ERRβ in Ovarian Cancer

The state of the data on ERRβ expression in OC appears to be even more deficient. A study at the mRNA level stated that ERRβ levels in the OC tissue were too low for retrieving reliable information [144]. In our IHC analysis, we were able to detect ERRβ in 82.2% of the included OC cases [145]. Higher expression of ERRβ in serous OCs was found to lead to a significantly decreased overall survival (*p* < 0.05) [145]. These very preliminary findings might suggest a tumor-promoting role of ERRβ in OC. This would be in line with a study reporting that in breast cancer, ERRβ was upregulated by estrogens in an ERα-dependent manner, and was found to be inversely correlated with overall survival of breast cancer patients [153]. In contrast, in prostate cancer cells, ERRβ was reported to suppress growth via p21(WAF1) induction, making it a potential therapeutic target in this cancer entity [154]. 

#### 4.1.4. Role of ERRγ in Ovarian Cancer

With regard to ERRγ, the progression-free survival of women with high ERRγ expressing OCs was reported to be longer in the small study presented above [143]. In our study analyzing 208 OC specimens, we detected ERRγ expression in more than 90% of all included OC cases [145]. We found that this ERR type exhibited the strongest impact on survival [145]. Women with serous OCs expressing low levels of ERRγ survived significantly longer than patients with high ERRγ-expressing tumors (*p* < 0.05) [145]. In a multivariable model, ERRγ was found to be an independent prognostic factor for overall survival of women with serous OC [145]. These data imply that this receptor affects OC development in a tumor-promoting manner. Future studies urgently need to elucidate the role of ERRγ not only in ovarian cancer, but also in other cancers, as it seems to play a more significant role in carcinogenesis than previously estimated.

Taken together, data on the role of ERRα in OC remain sparse. However, they suggest a promoting effect on carcinogenesis of this tumor entity. Regarding the role of ERRβ, and particularly ERRγ in OC, a tumor-promoting effect does seem possible. However, further studies investigating the role of all ERRs with higher numbers of included cases, especially with regard to distinct subtypes, are necessary, as this malignancy is characterized by a strong heterogeneity. 

### 4.2. ERRs in Endometrial Cancer

#### 4.2.1. ERRα Protein and mRNA Expression in Endometrial Cancer

Available data on the role of ERRs in EC is similarly scarce, as just described for OCs. In 2006, Watanabe et al. detected ERRα expression in human EC tissues by IHC and via RT-PCR in four EC cell lines (Ishikawa, Hec1a, KLE, and SNGII) and 11 human endometrial tissues [155]. In another study, Sun et al. showed that relative ERRα mRNA and protein levels were significantly higher in ERα-positive RL-952 and AN3-CA cells compared to ERα-negative HEC-1A and HEC-1B cells (*p* < 0.05) [156]. In a IHC-based analysis by Matisushima et al. including 50 EC samples, ERRα was found to be expressed in all the investigated specimens, and high expression levels were found to correlate with advanced stage and the serous subtype (*p* < 0.01) [157]. Similar data was obtained by another study, showing elevated ERRα mRNA levels and histoscores to be increased with the clinical stage and myometrial invasion, regardless of tumor grading [158]. Huang et al. investigated the role of ERRα and peroxisome proliferator-activated receptor-γ (PPARγ)—a key regulator in energy metabolism—in EC [159]. They identified a PPARγ/ERRα ratio of 1.86 or less to be an independent risk factor of EC (OR 14.85; 95% CI 1.6–137.75, *p* = 0.018) [159]. No difference in the overall or disease free survival was observed when comparing ERRα-positive and ERRα-negative patients [159]. When using PPARγ/ERRα as a predictor of prognosis, they found that overall and disease free survival were the lowest for PPARγ-negative and ERRα-positive patients (overall survival: 100.00% vs. 85.19%, *p* < 0.001; disease free survival: 100.00% vs. 77.78%, *p* < 0.001) [159]. In the subgroups being compared, EC patients with absent expression of PPARγ but with expression of ERRα, had the worst overall survival and disease-free survival rates (both *p* < 0.001) [159]. Taken together, these data support a tumor-promoting role of ERRα in EC.

#### 4.2.2. In Vitro Studies on ERRα Action in Endometrial Cancer

**Figure 1:**
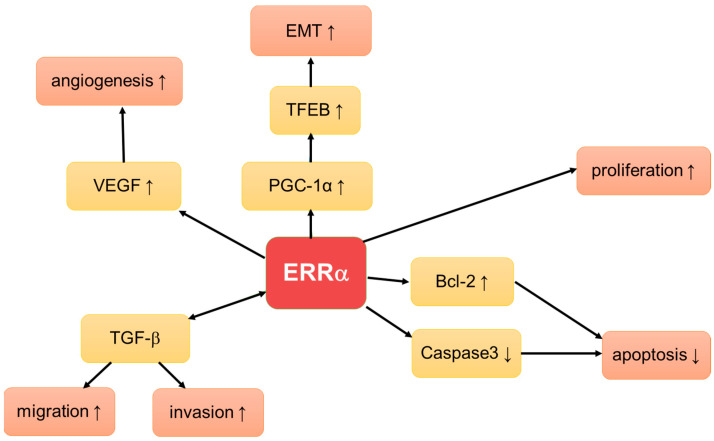
ERRα-induced tumor-promoting actions in EC cells (↑ increase; ↓ decrease).

The tumor-promoting role of ERRα in EC is supported by in vitro studies (Figure 1). In vitro data showed that ERRα knockdown by means of specific siRNA decreased proliferation and angiogenesis, and increased caspase3-induced apoptosis by induction of cell cycle arrest in the mitotic phase [157]. In bioinformatic analyses, PPARγ and ERRα were found to opposingly regulate the same genes involved in proliferation and apoptosis via the Bcl-2/Caspase3 pathway [159]. 

Peroxisome proliferator-activated receptor (PPAR) coactivator-1α (PGC-1α) acts as a coactivator of ERRα, and is among others involved in the regulation of cellular oxidative phosphorylation and liposome metabolism [160,161,162]. Overexpression of ERRα led to enhanced PGC-1α expression resulting in the increased activity of transcription factor EB TFEB, which is promoted in EC cells. This finding was supported by a recent publication by Mao et al. [160,163].

Yoriki et al. examined the impact of ERRα and PGC-1α on EMT in [164]. They transfected Ishikawa and HEC-1A EC cells with either ERRα/PGC-1α expression plasmids, or silenced them for ERRα expression and cultured them with telomerase-transformed human endometrial stromal cells (T-HESCs) [164]. Cells with a high expression of ERRα and PGC-1α showed increased levels of the EMT-associated proteins vimentin, Snail, and the zinc finger E-box binding homeobox 1 (ZEB1), while in T-HESCs, the expression of the transforming growth factor-beta (TGF-β) was increased [164]. These effects were subsequently reversed by ERRα knockdown [164]. Furthermore, TGF-β-induced migration and invasion were abrogated by ERRα knockdown. Together, these data indicate that in EC TGF-β-induced EMT could be inhibited by targeting ERRα via interactions between stroma and cancer [164]. The role of TGF-β in ERRα-mediated actions in EC was also investigated by Huang et al. [165]. In their study, expression levels of ERRα, but not ERRβ or ERRγ, were significantly increased in EC cells and tissues compared to healthy controls. They used thirty-two tumor samples that were paired with adjacent normal endometrial tissues and six EC cell lines [165]. Targeted inhibition of ERRα by siRNA or its inverse agonist XCT-790 suppressed the migration and invasion of the EC cells, and led to a decreased expression of TGF-β [165]. The latter was explained by ERRα directly binding to the promoter of the *TGFB1* gene [165]. Previous studies indicated that TGF-β can positively regulate its own expression in normal and transformed cells. Huang et al. showed that exogenous TGF-β increased not only the mRNA and protein levels of TGF-β, but also of ERRα in both HEC1A and ECC cells [165]. These effects were abolished by the knockdown of ERRα using specific siRNA [165]. They observed that targeted inhibition of ERRα/ TGF-β was able to suppress the invasion of EC cells in vitro synergistically [165]. Thus, the authors suggested that ERRα could trigger cell migration and invasion via an increase in the positive feedback regulation of TGF-β [165].

The inverse ERRα agonist XCT790 was shown to suppress colony formation and cell proliferation in a dose- and time-dependent manner (*p* < 0.01), and to induce apoptosis as well as cell cycle arrest in the mitotic phase [166]. XCT790 triggered growth inhibition and apoptosis in EC cell lines independently of ERα [156]. In vivo studies reported a significant decrease in tumor growth and angiogenesis using XCT790 in xenograft models (*p* < 0.01) [166]. The same effect was observed in ERRα knockdown xenografts (*p* < 0.01) [157].

Contrary information was provided by the following studies [155,167]: Sun et al., stably overexpressed ERRα in ER-responsive Ishikawa EC cells, and ER-nonresponsive HEC-1A cells [167]. They found that overexpression of ERRα inhibited cell proliferation in ERα-responsive Ishikawa cells, and stimulated cell proliferation in the ERα-nonresponsive HEC-1A cells. In this study, overexpression of ERRα repressed the mRNA level of ERα, but not ERβ, indicating a cross-talk between ERRα and ERα [167]. In line with this, in the report published by Watanabe et al., overexpression of ERRα resulted in a decreased proliferation of the ERα-responsive Ishikawa EC cells [155]. The authors suggested that endogenous ERRα suppresses the ERα-dependent estrogen-induced and ERE-dependent transcriptional activities in EC and ERRα to operate as either an activator or repressor of ERE-dependent transcription based upon other properties of the cell [155].

#### 4.2.3. ERRγ Expression in Endometrial Cancer

In comparison to ERRα, data on the role of ERRγ in EC is limited. Tong et al. recently tried to address this topic using data from three different databases: the Cancer Genome Atlas (TCGA), the Clinical Proteomic Tumor Analysis Consortium (CPTAC) databases, and the International Cancer Genome Consortium (ICGC) databases [168]. They included tissue samples of 525 EC patients and 35 normal endometrium specimens [168]. ERRγ protein expression was notably higher in EC tissues compared to normal endometrium (*p* < 0.001), which was consistent with the result obtained by the analysis of the TCGA data, suggesting a tumor-promoting role of ERRγ in EC [168]. When investigating data from the ICGC database, the overall and disease-free survival were not dependent on ERRγ mutations (*p* > 0.05) [168]. Moreover, survival of 79 women with EC did not differ when comparing tumors with different ERRγ protein expression levels [168].

#### 4.2.4. In Vitro Studies on ERRγ Action in Endometrial Cancer

In line with this, stable overexpression of ERRγupregulated cell growth of Ishikawa and HEC1A EC cell lines in vitro. In contrast, cell growth in ERα-positive EC cells decreased after treatment with DY131, a selective ERRγ agonist, whereas the effect was reversed in ERα-negative cells, suggesting that the impact of ERRγ on carcinogenesis of EC is ERα-dependent [169]. However, further studies are necessary to elucidate the underlying mechanisms. 

As already mentioned above, abnormal glucose metabolism is a frequent feature observed in patients with EC [168,170]. Several studies suggest hyperglycemia to be associated with a shorter survival of EC patients [168,171,172]. ERRγ was shown to regulate numerous key enzymes in cell glucose, lipid, and amino acid metabolism, and has been linked to abnormal gluconeogenesis and insulin resistance, among others [168,173,174]. Moreover, ERRγ was highly expressed in diabetic patients with poor blood glucose control [168,175]. Tong et al. found the overexpression of ERRγ to be significantly associated with the deep myometrial invasion of EC (*p* = 0.004). Moreover, patients with ECs that were deeply infiltrating the myometrium had higher fasting blood glucose levels compared to those with superficial myometrial invasion (*p* = 0.040), and serum ERRγ levels positively correlated with fasting glucose levels [168].

In summary, the data available to date suggest a tumor-promoting role of ERRα and ERRγ in EC. In contrast, the function of ERRβ in this tumor entity remains obscure, as no conclusive studies were available. However, in vivo studies are necessary before an implication in the clinical setting seems feasible. 

## 5. Conclusions

In conclusion, current data strongly suggest ERβ to function as a tumor suppressor in OC. In this cancer entity, the expression of ERβ was shown to be significantly lower compared to normal ovarian tissue. Moreover, the overall, and progression-free survival of OC patients was significantly longer when their tumors expressed elevated levels of ERβ compared to those with a lower ERβ expression. In vitro overexpression of ERβ leads to reduced proliferation, motility, and migration, as well as increased rates of OC cell apoptosis. This action could be evoked by the use of specific ERβ agonists. Some of them were shown to limit CSC, as well as reduce the risk of metastasis and relapse using this quality. Regulatory processes considering the subcellular location of ERβ must be taken into account in future studies. Regarding the role of different ERβ splice variants, present studies suggest that ERβ2 and ERβ5 might function as tumor promotors in OC. However, further attempts are needed to confirm these data, and to elucidate the role of other splice variants in OC. To better understand the distinct roles of ERβ in different OC subtypes, further studies including larger patients’ cohorts are required. 

In EC, the role of ERβ remains inconclusive. Whereas in vitro studies demonstrated a growth-inhibitory function of this receptor in EC cell lines, several studies analyzing the expression of ERβ in EC tissues came to conflicting results. Several mRNA-based and IHC studies suggested a tumor-suppressive role of this receptor in EC, but others came to contradictory results. Various IHC studies on ERβ expression are now known to have used non-specific antibodies, which is one explanation for the inconsistent results observed from these studies. Furthermore, the in-part contradictory data on the role of ERβ in EC tissue might be due to the distinct functions of ERβ depending on the ERα/ERβ ratio. It was speculated that in healthy, or only minimally dedifferentiated endometrial tissue, with the expression of the full complement of hormone receptors, ERβ might act as a tumor-suppressor [107]. In completely dedifferentiated, high-grade EC, with the loss of other hormone receptor subtypes, ERβ might exert a tumor-promoting function [107]. However, considering all studies available, further efforts are needed to examine to what extent the consistent results from in vitro studies can be verified on the tissue level by correlation of ERβ expression and of the ERα/ERβ ratio in EC tissue with patients’ survival.

To date, studies examining the role of GPER1 in OC came to conflicting results, suggesting either tumor-suppressive or -promoting functions. Given that GPER1 function is affected by various pathways, its function in OC might depend on the activation status of these pathways in OC cells. Furthermore, the expression of ERα and ERβ was known to modulate GPER1 effects. Therefore, in the future, these interactions and the molecular characteristics of the employed cell lines and tissue samples, including the expression of nuclear ERs should be taken into account more closely when investigating the role of GPER1 in OC. Further studies correlating GPER1 protein expression with patient survival, as well as in vivo studies, are needed to elucidate the role of this receptor in OC.

With regard to EC, both in vitro and tissue studies on the role of GPER1 came to conflictive results. More studies, including such correlating GPER1 protein expression in EC tissue with patient survival, and in vivo approaches examining the effects of GPER1 modulation with specific antagonists in animal models will be necessary to elucidate the role of GPER1 in this cancer entity. 

The current data on the role of ERRα in OC suggests a tumor-promoting role in this tumor entity. Despite some controversies, published data suggests higher expression levels of ERRα in late-stage and high-grade OCs compared to early-stage and low-grade tissues [146]. In vitro, ERRα not only induces motility and invasion of OC cells, but also increases EMT. Regarding the function of ERRβ and ERRγ in OC, the limited data available might also suggest a tumor-promoting action. However, further data investigating the role of all ERRs with higher numbers of included cases especially with regard to distinct subtypes are necessary, as this malignancy is characterized by a strong heterogeneity. In EC, the available data suggests a tumor-promoting role of ERRα and ERRγ. Among others, ERRα is regulated by TGF-β, leading to an induction of EMT. Moreover, ERRα expression is associated with an increase in proliferation and angiogenesis, as well as a decrease in apoptosis. The impact of ERRβ in this tumor entity remains obscure, as no conclusive studies are available. However, larger studies correlating ERR expression in EC tissue with patient survival and in vivo studies on animal models are necessary to further elucidate the role of ERRs in this cancer entity. Overall, the new molecular classification of EC provides further insights into the different courses of this disease. The extent to which ERβ, GPER1, and ERRs are associated with this classification, and their relevance for the efficacy of new therapeutic options in EC such as checkpoint inhibitors and tyrosine kinase inhibitors, must be evaluated in future research.

## References

[1] Chen G.G., Zeng Q., Tse G.M. (2008). Estrogen and its receptors in cancer. Med. Res. Rev..

[2] Zhao C., Dahlman-Wright K., Gustafsson J.-Å. (2010). Estrogen Signaling via Estrogen Receptor β. J. Biol. Chem..

[3] Crandall D.L., Busler D.E., Novak T.J., Weber R.V., Kral J.G. (1998). Identification of Estrogen Receptor β RNA in Human Breast and Abdominal Subcutaneous Adipose Tissue. Biochem. Biophys. Res. Commun..

[4] Kuiper G.G., Shughrue P.J., Merchenthaler I., Gustafsson J. (1998). The Estrogen Receptor β Subtype: A Novel Mediator of Estrogen Action in Neuroendocrine Systems. Front. Neuroendocr..

[5] Balla B., Sárvári M., Kósa J.P., Kocsis-Deák B., Tobiás B., Árvai K., Takács I., Podani J., Liposits Z., Lakatos P. (2019). Long-term selective estrogen receptor-beta agonist treatment modulates gene expression in bone and bone marrow of ovariectomized rats. J. Steroid Biochem. Mol. Biol..

[6] Liu J.Y.H., Lin G., Fang M., Rudd J.A. (2019). Localization of estrogen receptor ERα, ERβ and GPR30 on myenteric neurons of the gastrointestinal tract and their role in motility. Gen. Comp. Endocrinol..

[7] Tamer S.A., Yıldırım A., Arabacı Ş., Çiftçi S., Akın S., Sarı E., Köroğlu M.K., Ercan F., Yüksel M., Çevik Ö. (2019). Treatment with estrogen receptor agonist ERβ improves torsion-induced oxidative testis injury in rats. Life Sci..

[8] Le Moëne O., Stavarache M., Ogawa S., Musatov S., Ågmo A. (2019). Estrogen receptors α and β in the central amygdala and the ventromedial nucleus of the hypothalamus: Sociosexual behaviors, fear and arousal in female rats during emotionally challenging events. Behav. Brain Res..

[9] Kim H., Park J., Leem H., Cho M., Yoon J.-H., Maeng H.-J., Lee Y. (2019). Rhododendrin-Induced RNF146 Expression via Estrogen Receptor β Activation is Cytoprotective Against 6-OHDA-Induced Oxidative Stress. Int. J. Mol. Sci..

[10] Han S.J., Jung S.Y., Wu S.-P., Hawkins S.M., Park M.J., Kyo S., Qin J., Lydon J.P., Tsai S.Y., Tsai M.-J. (2015). Estrogen Receptor β Modulates Apoptosis Complexes and the Inflammasome to Drive the Pathogenesis of Endometriosis. Cell.

[11] Du M., Shan J., Feng A., Schmull S., Gu J., Xue S. (2020). Oestrogen Receptor β Activation Protects Against Myocardial Infarction via Notch1 Signalling. Cardiovasc. Drugs Ther..

[12] Ofosu W.A., Mohamed D., Corcoran O., Ojo O.O. (2019). The Role of Oestrogen Receptor Beta (ERβ) in the Aetiology and Treatment of Type 2 Diabetes Mellitus. Curr. Diabetes Rev..

[13] Bharathi C., Anupama D., Pratibha N., Venkateshwari A. (2019). Impact of Genetic Variants in Estrogen Receptor-β Gene in the Etiology of Uterine Leiomyomas. J. Reprod. Infertil..

[14] Lattrich C., Stegerer A., Häring J., Schüler S., Ortmann O., Treeck O. (2013). Estrogen receptor β agonists affect growth and gene expression of human breast cancer cell lines. Steroids.

[15] Schüler-Toprak S., Moehle C., Skrzypczak M., Ortmann O., Treeck O. (2017). Effect of estrogen receptor β agonists on proliferation and gene expression of ovarian cancer cells. BMC Cancer.

[16] Treeck O., Diepolder E., Skrzypczak M., Schüler-Toprak S., Ortmann O. (2019). Knockdown of estrogen receptor β increases proliferation and affects the transcriptome of endometrial adenocarcinoma cells. BMC Cancer.

[17] Majumdar S., Rinaldi J.C., Malhotra N.R., Xie L., Hu D.-P., Gauntner T.D., Grewal H.S., Hu W.-Y., Kim S.H., Katzenellenbogen J.A. (2019). Differential Actions of Estrogen Receptor α and β via Nongenomic Signaling in Human Prostate Stem and Progenitor Cells. Endocrinology.

[18] Ibrahim A., Hugerth L.W., Hases L., Saxena A., Seifert M., Thomas Q., Gustafsson J., Engstrand L., Williams C. (2019). Colitis-induced colorectal cancer and intestinal epithelial estrogen receptor beta impact gut microbiota diversity. Int. J. Cancer.

[19] Santti K., Ihalainen H., Rönty M., Msc C.K., Haglund C., Sampo M., Tarkkanen M., Blomqvist C. (2019). Estrogen receptor beta expression correlates with proliferation in desmoid tumors. J. Surg. Oncol..

[20] Liang L., Williams M.D., Bell D. (2019). Expression of PTEN, Androgen Receptor, HER2/neu, Cytokeratin 5/6, Estrogen Receptor-Beta, HMGA2, and PLAG1 in Salivary Duct Carcinoma. Head Neck Pathol..

[21] Zhou Y., Liu X. (2020). The role of estrogen receptor beta in breast cancer. Biomark. Res..

[22] Pujol P., Rey J.M., Nirde P., Roger P., Gastaldi M., Laffargue F., Rochefort H., Maudelonde T. (1998). Differential expression of estrogen receptor-alpha and -beta messenger RNAs as a potential marker of ovarian carcinogenesis. Cancer Res..

[23] Li A.J., Baldwin R.L., Karlan B.Y. (2003). Estrogen and progesterone receptor subtype expression in normal and malignant ovarian epithelial cell cultures. Am. J. Obstet. Gynecol..

[24] Matthews J., Gustafsson J.A. (2003). Estrogen Signaling: A Subtle Balance Between ER alpha and ER beta. Mol. Interv..

[25] Fujimuraa T., Takahashi S., Uranob T., Ogawab S., Ouchib Y., Kitamuraa T., Muramatsuc M., Inouebcd S. (2001). Differential Expression of Estrogen Receptor β (ERβ) and Its C-Terminal Truncated Splice Variant ERβcx as Prognostic Predictors in Human Prostatic Cancer. Biochem. Biophys. Res. Commun..

[26] Park B.-W., Kim K.-S., Heo M.-K., Yang W.-I., Kim S.I., Kim J.-H., Kim G.E., Lee K.S. (2006). The changes of estrogen receptor-β variants expression in breast carcinogenesis: Decrease of estrogen receptor-β2 expression is the key event in breast cancer development. J. Surg. Oncol..

[27] Esslimani-Sahla M., Simony-Lafontaine J., Kramar A., Lavaill R., Mollevi C., Warner M., Gustafsson J.-A., Rochefort H. (2004). Estrogen Receptor β (ERβ) Level but Not Its ERβcx Variant Helps to Predict Tamoxifen Resistance in Breast Cancer. Clin. Cancer Res..

[28] Häring J., Skrzypczak M., Stegerer A., Lattrich C., Weber F., Görse R., Ortmann O., Treeck O. (2012). Estrogen receptor β transcript variants associate with oncogene expression in endometrial cancer. Int. J. Mol. Med..

[29] Treeck O., Pfeiler G., Mitter D., Lattrich C., Piendl G., Ortmann O. (2007). Estrogen receptor β1 exerts antitumoral effects on SK-OV-3 ovarian cancer cells. J. Endocrinol..

[30] Springwald A., Lattrich C., Skrzypczak M., Goerse R., Ortmann O., Treeck O. (2010). Identification of novel transcript variants of estrogen receptor α, β and progesterone receptor gene in human endometrium. Endocrine.

[31] Zhou R., Treeck O., Horn F., Ortmann O. (2005). Effects of prolonged tamoxifen treatment on receptor expression and apoptosis of ovarian cancer cells. Gynecol. Oncol..

[32] Häring J., Schüler S., Lattrich C., Ortmann O., Treeck O. (2012). Role of estrogen receptor β in gynecological cancer. Gynecol. Oncol..

[33] Hernández-Silva C.D., Villegas-Pineda J.C., Pereira-Suárez A.L. (2020). Expression and Role of the G Protein-Coupled Estrogen Receptor (GPR30/GPER) in the Development and Immune Response in Female Reproductive Cancers. Front. Endocrinol..

[34] Tanida T. (2022). Molecular dynamics of estrogen-related receptors and their regulatory proteins: Roles in transcriptional control for endocrine and metabolic signaling. Anat. Sci. Int..

[35] Crevet L., Vanacker J.-M. (2020). Regulation of the expression of the estrogen related receptors (ERRs). Cell Mol. Life Sci..

[36] Yeramian A., Moreno-Bueno G., Dolcet X., Catasus L., Abal M., Colas E., Matias-Guiu X. (2012). Endometrial carcinoma: Molecular alterations involved in tumor development and progression. Oncogene.

[37] Sivridis E., Giatromanolaki A. (2011). The pathogenesis of endometrial carcinomas at menopause: Facts and figures. J. Clin. Pathol..

[38] Šmuc T., Rižner T.L. (2009). Aberrant pre-receptor regulation of estrogen and progesterone action in endometrial cancer. Mol. Cell Endocrinol..

[39] Collins F., MacPherson S., Brown P., Bombail V., Williams A.R., Anderson R.A., Jabbour H.N., Saunders P.T. (2009). Expression of oestrogen receptors, ERα, ERβ, and ERβ variants, in endometrial cancers and evidence that prostaglandin F may play a role in regulating expression of ERα. BMC Cancer.

[40] Bokhman J.V. (1983). Two pathogenetic types of endometrial carcinoma. Gynecol. Oncol..

[41] Kandoth C., Schultz N., Cherniack A.D., Akbani R., Liu Y., Shen H., Robertson A.G., Pashtan I., Shen R., Benz C.C. (2013). Integrated genomic characterization of endometrial carcinoma. Nature.

[42] WHO Classification of Tumours Editorial Board (2020). Female Genital Tumours.

[43] Fixemer T., Remberger K., Bonkhoff H. (2003). Differential expression of the estrogen receptor beta (ERbeta) in human prostate tissue, premalignant changes, and in primary, metastatic, and recurrent prostatic adenocarcinoma. Prostate.

[44] Foley E.F., Jazaeri A.A., Shupnik M.A., Jazaeri O., Rice L.W. (2000). Selective loss of estrogen receptor beta in malignant human colon. Cancer Res..

[45] Park B.-W., Kim K.-S., Heo M.-K., Ko S.-S., Lee K.S., Hong S., Yang W.-I., Kim J.-H., Kim G.E. (2003). Expression of Estrogen Receptor-β in Normal Mammary and Tumor Tissues: Is it Protective in Breast Carcinogenesis?. Breast Cancer Res. Treat..

[46] Roger P., Sahla M.E., Mäkelä S., Gustafsson J.A., Baldet P., Rochefort H. (2001). Decreased expression of estrogen receptor beta protein in proliferative preinvasive mammary tumors. Cancer Res..

[47] Skliris G.P., Munot K., Bell S.M., Carder P.J., Lane S., Horgan K., Lansdown M.R.J., Parkes A.T., Hanby A.M., Markham A.F. (2003). Reduced expression of oestrogen receptor beta in invasive breast cancer and its re-expression using DNA methyl transferase inhibitors in a cell line model. J. Pathol..

[48] Brandenberger (1998). Estrogen receptor alpha (ER-alpha) and beta (ER-beta) mRNAs in normal ovary, ovarian serous cystadenocarcinoma and ovarian cancer cell lines: Down-regulation of ER-beta in neoplastic tissues. J. Clin. Endocrinol. Metab..

[49] Chan K.K.L., Wei N., Liu S.S., Xiao-Yun L., Cheung A.N., Ngan H.Y.S. (2008). Estrogen receptor subtypes in ovarian cancer: A clinical correlation. Obstet. Gynecol..

[50] De Stefano I., Zannoni G.F., Prisco M.G., Fagotti A., Tortorella L., Vizzielli G., Mencaglia L., Scambia G., Gallo D. (2011). Cytoplasmic expression of estrogen receptor beta (ERβ) predicts poor clinical outcome in advanced serous ovarian cancer. Gynecol. Oncol..

[51] Rutherford T., Brown W.D., Sapi E., Aschkenazi S., Muñoz A., Mor G. (2000). Absence of estrogen receptor-beta expression in metastatic ovarian cancer. Obstet. Gynecol..

[52] Suzuki F., Akahira J.-I., Miura I., Suzuki T., Ito K., Hayashi S.-I., Sasano H., Yaegashi N. (2008). Loss of estrogen receptor β isoform expression and its correlation with aberrant DNA methylation of the 5′-untranslated region in human epithelial ovarian carcinoma. Cancer Sci..

[53] Filigheddu N., Sampietro S., Chianale F., Porporato P.E., Gaggianesi M., Gregnanin I., Rainero E., Ferrara M., Perego B., Riboni F. (2011). Diacylglycerol kinase α mediates 17-β-estradiol-induced proliferation, motility, and anchorage-independent growth of Hec-1A endometrial cancer cell line through the G protein-coupled estrogen receptor GPR30. Cell Signal..

[54] Lindgren P.R., Cajander S., Bäckström T., Gustafsson J., Mäkelä S., Olofsson J.I. (2004). Estrogen and progesterone receptors in ovarian epithelial tumors. Mol. Cell Endocrinol..

[55] Halon A., Nowak-Markwitz E., Maciejczyk A., Pudelko M., Gansukh T., Györffy B., Donizy P., Murawa D., Matkowski R., Spaczynski M. (2011). Loss of estrogen receptor beta expression correlates with shorter overall survival and lack of clinical response to chemotherapy in ovarian cancer patients. Anticancer Res..

[56] Hyder S.M., Chiappetta C., Stancel G.M. (1999). Interaction of human estrogen receptors α and β with the same naturally occurring estrogen response elements. Biochem. Pharmacol..

[57] Bogush T.A., Basharina A.A., Bogush E.A., Ryabinina O.M., Tjulandina A.S., Tjulandin S.A. (2018). Estrogen Receptors alpha and beta in Ovarian Cancer: Expression Level and Prognosis. Dokl. Biochem. Biophys..

[58] Sieh W., Köbel M., Longacre T.A., Bowtell D.D., Defazio A., Goodman M.T., Høgdall E., Deen S., Wentzensen N., Moysich K.B. (2013). Hormone-receptor expression and ovarian cancer survival: An Ovarian Tumor Tissue Analysis consortium study. Lancet Oncol..

[59] Kumar S., Dasari P., Sylvia M.T. (2012). The expression of immunohistochemical markers estrogen receptor, progesterone receptor, Her-2-neu, p53 and Ki-67 in epithelial ovarian tumors and its correlation with clinicopathologic variables. Indian J. Pathol. Microbiol..

[60] Damião R.D.S., Oshima C.F., Stávale J., Gonçalves W. (2007). Analysis of the expression of estrogen receptor, progesterone receptor and chicken ovalbumin upstream promoter-transcription factor I in ovarian epithelial cancers and normal ovaries. Oncol. Rep..

[61] Tarallo R., Giurato G., Bruno G., Ravo M., Rizzo F., Salvati A., Ricciardi L., Marchese G., Cordella A., Rocco T. (2017). The nuclear receptor ERβ engages AGO2 in regulation of gene transcription, RNA splicing and RISC loading. Genome Biol..

[62] Schüler-Toprak S., Weber F., Skrzypczak M., Ortmann O., Treeck O. (2018). Estrogen receptor β is associated with expression of cancer associated genes and survival in ovarian cancer. BMC Cancer.

[63] Shafrir A.L., Babic A., Kuliszewski M.G., Rice M.S., Townsend M.K., Hecht J.L., Tworoger S.S. (2020). Estrogen Receptor-β Expression of Ovarian Tumors and Its Association with Ovarian Cancer Risk Factors. Cancer Epidemiol. Biomark. Prev..

[64] Oturkar C.C., Gandhi N., Rao P., Eng K.H., Miller A., Singh P.K., Zsiros E., Odunsi K.O., Das G.M. (2022). Estrogen Receptor-Beta2 (ERβ2)–Mutant p53–FOXM1 Axis: A Novel Driver of Proliferation, Chemoresistance, and Disease Progression in High Grade Serous Ovarian Cancer (HGSOC). Cancers.

[65] Ciucci A., Zannoni G.F., Travaglia D., Petrillo M., Scambia G., Gallo D. (2014). Prognostic significance of the estrogen receptor beta (ERβ) isoforms ERβ1, ERβ2, and ERβ5 in advanced serous ovarian cancer. Gynecol. Oncol..

[66] Chan K.K.L., Siu M.K.Y., Jiang Y.X., Wang J.J., Wang Y., Leung T.H.Y., Liu S.S., Cheung A.N.Y., Ngan H.Y.S. (2017). Differential expression of estrogen receptor subtypes and variants in ovarian cancer: Effects on cell invasion, proliferation and prognosis. BMC Cancer.

[67] Warner M., Fan X., Strom A., Wu W., Gustafsson J.-Å. (2021). 25 years of ERβ: A personal journey. J. Mol. Endocrinol..

[68] Nilsson S., Mäkelä S., Treuter E., Tujague M., Thomsen J., Andersson G., Enmark E., Pettersson K., Warner M., Gustafsson J.A. (2001). Mechanisms of Estrogen Action. Physiol. Rev..

[69] Paruthiyil S., Parmar H., Kerekatte V., Cunha G.R., Firestone G.L., Leitman D.C. (2004). Estrogen Receptor β Inhibits Human Breast Cancer Cell Proliferation and Tumor Formation by Causing a G2 Cell Cycle Arrest. Cancer Res..

[70] Ström A., Hartman J., Foster J.S., Kietz S., Wimalasena J., Gustafsson J.-A. (2004). Estrogen receptor β inhibits 17β-estradiol-stimulated proliferation of the breast cancer cell line T47D. Proc. Natl. Acad. Sci. USA.

[71] Hayashido Y., Lucas A., Rougeot C., Godyna S., Argraves W.S., Rochefort H. (1998). Estradiol and fibulin-1 inhibit motility of human ovarian-and breast-cancer cells induced by fibronectin. Int. J. Cancer.

[72] Treeck O., Pfeiler G., Horn F., Federhofer B., Houlihan H., Vollmer A., Ortmann O. (2007). Novel estrogen receptor beta transcript variants identified in human breast cancer cells affect cell growth and apoptosis of COS-1 cells. Mol. Cell Endocrinol..

[73] Liu M.-M., Albanese C., Anderson C.M., Hilty K., Webb P., Uht R.M., Price R.H., Pestell R.G., Kushner P.J. (2002). Opposing Action of Estrogen Receptors α and β on Cyclin D1 Gene Expression. J. Biol. Chem..

[74] Lazennec G. (2006). Estrogen receptor beta, a possible tumor suppressor involved in ovarian carcinogenesis. Cancer Lett..

[75] Planas-Silva M.D., Weinberg R.A. (1997). Estrogen-Dependent Cyclin E-cdk2 Activation through p21 Redistribution. Mol. Cell Biol..

[76] Worsley S.D., Ponder B.A., Davies B.R. (1997). Overexpression of Cyclin D1 in Epithelial Ovarian Cancers. Gynecol. Oncol..

[77] Banerjee A., Cai S., Xie G., Li N., Bai X., Lavudi K., Wang K., Zhang X., Zhang J., Patnaik S. (2022). A Novel Estrogen Receptor β Agonist Diminishes Ovarian Cancer Stem Cells via Suppressing the Epithelial-to-Mesenchymal Transition. Cancers.

[78] Prasad S., Ramachandran S., Gupta N., Kaushik I., Srivastava S.K. (2020). Cancer cells stemness: A doorstep to targeted therapy. Biochim. Biophys. Acta BBA Mol. Basis Dis..

[79] He Y., Alejo S., Venkata P.P., Johnson J.D., Loeffel I., Pratap U.P., Zou Y., Lai Z., Tekmal R.R., Kost E.R. (2022). Therapeutic Targeting of Ovarian Cancer Stem Cells Using Estrogen Receptor Beta Agonist. Int. J. Mol. Sci..

[80] Hu L., McArthur C., Jaffe R.B. (2010). Ovarian cancer stem-like side-population cells are tumourigenic and chemoresistant. Br. J. Cancer.

[81] Zong X., Nephew K.P. (2019). Ovarian Cancer Stem Cells: Role in Metastasis and Opportunity for Therapeutic Targeting. Cancers.

[82] Zhang S., Balch C., Chan M.W., Lai H.-C., Matei D., Schilder J.M., Yan P.S., Huang T.H.-M., Nephew K.P. (2008). Identification and Characterization of Ovarian Cancer-Initiating Cells from Primary Human Tumors. Cancer Res..

[83] Keyvani V., Farshchian M., Esmaeili S.-A., Yari H., Moghbeli M., Nezhad S.-R.K., Abbaszadegan M.R. (2019). Ovarian cancer stem cells and targeted therapy. J. Ovarian Res..

[84] Schüler S., Lattrich C., Skrzypczak M., Fehm T., Ortmann O., Treeck O. (2014). Polymorphisms in the promoter region of ESR2 gene and susceptibility to ovarian cancer. Gene.

[85] Liu Z., Kuokkanen S., Pal L. (2010). Steroid Hormone Receptor Profile of Premenopausal Endometrial Polyps. Reprod. Sci..

[86] Saegusa M., Okayasu I. (2000). Changes in Expression of Estrogen Receptors α and β in Relation to Progesterone Receptor and pS2 Status in Normal and Malignant Endometrium. Jpn. J. Cancer Res..

[87] Hojnik M., Sinreih M., Anko M., Hevir-Kene N., Knific T., Pirš B., Grazio S.F., Rižner T.L. (2023). The Co-Expression of Estrogen Receptors ERα, ERβ, and GPER in Endometrial Cancer. Int. J. Mol. Sci..

[88] Hu G., Zhang J., Zhou X., Liu J., Wang Q., Zhang B. (2020). Roles of estrogen receptor α and β in the regulation of proliferation in endometrial carcinoma. Pathol. Res. Pract..

[89] Obata T., Nakamura M., Mizumoto Y., Iizuka T., Ono M., Terakawa J., Daikoku T., Fujiwara H. (2017). Dual expression of immunoreactive estrogen receptor β and p53 is a potential predictor of regional lymph node metastasis and postoperative recurrence in endometrial endometrioid carcinoma. PLoS ONE.

[90] Chakravarty D., Srinivasan R., Ghosh S., Gopalan S., Rajwanshi A., Majumdar S. (2007). Estrogen receptor beta1 and the beta2/betacx isoforms in nonneoplastic endometrium and in endometrioid carcinoma. Int. J. Gynecol. Cancer.

[91] Skrzypczak M., Bieche I., Szymczak S., Tozlu S., Lewandowski S.A., Girault I., Radwanska K., Szczylik C., Jakowicki J.A., Lidereau R. (2004). Evaluation of mRNA expression of estrogen receptor beta and its isoforms in human normal and neoplastic endometrium. Int. J. Cancer.

[92] Leygue E., Dotzlaw H., Watson P.H., Murphy L.C. (1999). Expression of estrogen receptor beta1, beta2, and beta5 messenger RNAs in human breast tissue. Cancer Res..

[93] Collins F., Itani N., Esnal-Zufiaurre A., Gibson D.A., Fitzgerald C., Saunders P.T.K. (2020). The ERβ5 splice variant increases oestrogen responsiveness of ERαpos Ishikawa cells. Endocr. Relat. Cancer.

[94] Mylonas I., Jeschke U., Shabani N., Kuhn C., Kriegel S., Kupka M.S., Friese K. (2005). Normal and malignant human endometrium express immunohistochemically estrogen receptor alpha (ER-alpha), estrogen receptor beta (ER-beta) and progesterone receptor (PR). Anticancer. Res..

[95] Hu K., Zhong G., He F. (2005). Expression of estrogen receptors ERalpha and ERbeta in endometrial hyperplasia and adenocarcinoma. Int. J. Gynecol. Cancer.

[96] Mylonas I. (2010). Prognostic significance and clinical importance of estrogen receptor α and β in human endometrioid adenocarcinomas. Oncol. Rep..

[97] Jongen V., Briët J., de Jong R., Hoor K.T., Boezen M., van der Zee A., Nijman H., Hollema H. (2009). Expression of estrogen receptor-alpha and -beta and progesterone receptor-A and -B in a large cohort of patients with endometrioid endometrial cancer. Gynecol. Oncol..

[98] Zannoni G.F., Monterossi G., De Stefano I., Gargini A., Salerno M.G., Farulla I., Travaglia D., Vellone V.G., Scambia G., Gallo D. (2013). The expression ratios of estrogen receptor α (ERα) to estrogen receptor β1 (ERβ1) and ERα to ERβ2 identify poor clinical outcome in endometrioid endometrial cancer. Hum. Pathol..

[99] Takama F., Kanuma T., Wang D., Kagami I., Mizunuma H. (2001). Oestrogen receptor β expression and depth of myometrial invasion in human endometrial cancer. Br. J. Cancer.

[100] Fujimoto J., Sakaguchi H., Aoki I., Toyoki H., Tamaya T. (2002). Clinical Implications of the Expression of Estrogen Receptor-α and -β in Primary and Metastatic Lesions of Uterine Endometrial Cancers. Oncology.

[101] Shabani N., Kuhn C., Kunze S., Schulze S., Mayr D., Dian D., Gingelmaier A., Schindlbeck C., Willgeroth F., Sommer H. (2007). Prognostic significance of oestrogen receptor alpha (ERα) and beta (ERβ), progesterone receptor A (PR-A) and B (PR-B) in endometrial carcinomas. Eur. J. Cancer.

[102] Treeck O., Juhasz-Boess I., Lattrich C., Horn F., Goerse R., Ortmann O. (2008). Effects of exon-deleted estrogen receptor β transcript variants on growth, apoptosis and gene expression of human breast cancer cell lines. Breast Cancer Res. Treat..

[103] Treeck O., Lattrich C., Springwald A., Ortmann O. (2010). Estrogen receptor beta exerts growth-inhibitory effects on human mammary epithelial cells. Breast Cancer Res. Treat..

[104] Schüler-Toprak S., Häring J., Inwald E.C., Moehle C., Ortmann O., Treeck O. (2016). Agonists and knockdown of estrogen receptor β differentially affect invasion of triple-negative breast cancer cells in vitro. BMC Cancer.

[105] Cantrell L.A., Zhou C., Mendivil A., Malloy K.M., Gehrig P.A., Bae-Jump V.L. (2010). Metformin is a potent inhibitor of endometrial cancer cell proliferation—Implications for a novel treatment strategy. Gynecol. Oncol..

[106] Zhang J., Xu H., Zhou X., Li Y., Liu T., Yin X., Zhang B. (2017). Role of metformin in inhibiting estrogen-induced proliferation and regulating ERα and ERβ expression in human endometrial cancer cells. Oncol. Lett..

[107] Hapangama D.K., Kamal A.M., Bulmer J.N. (2015). Estrogen receptor β: The guardian of the endometrium. Hum. Reprod. Update.

[108] Jarzabek K., Koda M., Walentowicz-Sadlecka M., Grabiec M., Laudanski P., Wolczynski S. (2013). Altered expression of ERs, aromatase, and COX2 connected to estrogen action in type 1 endometrial cancer biology. Tumor Biol..

[109] Knapp P., Chabowski A., Błachnio-Zabielska A., Walentowicz-Sadłecka M., Grabiec M., Knapp P.A. (2013). Expression of Estrogen Receptors (α, β), Cyclooxygenase-2 and Aromatase in normal endometrium and endometrioid cancer of uterus. Adv. Med. Sci..

[110] Kolkova Z., Casslén V., Henic E., Ahmadi S., Ehinger A., Jirström K., Casslén B. (2012). The G protein-coupled estrogen receptor 1 (GPER/GPR30) does not predict survival in patients with ovarian cancer. J. Ovarian Res..

[111] Fujiwara S., Terai Y., Kawaguchi H., Takai M., Yoo S., Tanaka Y., Tanaka T., Tsunetoh S., Sasaki H., Kanemura M. (2012). GPR30 regulates the EGFR-Akt cascade and predicts lower survival in patients with ovarian cancer. J. Ovarian Res..

[112] Smith H.O., Arias-Pulido H., Kuo D.Y., Howard T., Qualls C.R., Lee S.-J., Verschraegen C., Hathaway H.J., Joste N.E., Prossnitz E. (2009). GPR30 predicts poor survival for ovarian cancer. Gynecol. Oncol..

[113] Yan Y., Liu H., Wen H., Jiang X., Cao X., Zhang G., Liu G. (2013). The novel estrogen receptor GPER regulates the migration and invasion of ovarian cancer cells. Mol. Cell Biochem..

[114] Ignatov T., Modl S., Thulig M., Weißenborn C., Treeck O., Ortmann O., Zenclussen A., Costa S.D., Kalinski T., Ignatov A. (2013). GPER-1 acts as a tumor suppressor in ovarian cancer. J. Ovarian Res..

[115] Schüler-Toprak S., Skrzypczak M., Ignatov T., Ignatov A., Ortmann O., Treeck O. (2020). G protein-coupled estrogen receptor 1 (GPER-1) and agonist G-1 inhibit growth of ovarian cancer cells by activation of anti-tumoral transcriptome responses: Impact of GPER-1 mRNA on survival. J. Cancer Res. Clin. Oncol..

[116] Fraungruber P., Kaltofen T., Heublein S., Kuhn C., Mayr D., Burges A., Mahner S., Rathert P., Jeschke U., Trillsch F. (2021). G Protein-Coupled Estrogen Receptor Correlates with Dkk2 Expression and Has Prognostic Impact in Ovarian Cancer Patients. Front. Endocrinol..

[117] Revankar C.M., Cimino D.F., Sklar L.A., Arterburn J.B., Prossnitz E.R. (2005). A Transmembrane Intracellular Estrogen Receptor Mediates Rapid Cell Signaling. Science.

[118] Sandén C., Broselid S., Cornmark L., Andersson K., Daszkiewicz-Nilsson J., Mårtensson U.E.A., Olde B., Leeb-Lundberg L.M.F. (2011). G Protein-Coupled Estrogen Receptor 1/G Protein-Coupled Receptor 30 Localizes in the Plasma Membrane and Traffics Intracellularly on Cytokeratin Intermediate Filaments. Mol. Pharmacol..

[119] Kolkova Z., Noskova V., Ehinger A., Hansson S., Casslén B. (2010). G protein-coupled estrogen receptor 1 (GPER, GPR 30) in normal human endometrium and early pregnancy decidua. Mol. Hum. Reprod..

[120] Gao F., Ma X., Ostmann A.B., Das S.K. (2011). GPR30 Activation Opposes Estrogen-Dependent Uterine Growth via Inhibition of Stromal ERK1/2 and Estrogen Receptor Alpha (ERα) Phosphorylation Signals. Endocrinology.

[121] Smith H.O., Leslie K., Singh M., Qualls C.R., Revankar C.M., Joste N.E., Prossnitz E. (2007). GPR30: A novel indicator of poor survival for endometrial carcinoma. Am. J. Obstet. Gynecol..

[122] Osaku D., Oishi T., Kawamura N., Iida Y., Komatsu H., Kudoh A., Chikumi J., Sato S., Harada T. (2021). Differential expression of estrogen receptor subtypes in ovarian high-grade serous carcinoma and clear cell carcinoma. Reprod. Med. Biol..

[123] Zhu C.-X., Xiong W., Wang M.-L., Yang J., Shi H.-J., Chen H.-Q., Niu G. (2018). Nuclear G protein-coupled oestrogen receptor (GPR30) predicts poor survival in patients with ovarian cancer. J. Int. Med. Res..

[124] Wang C., Lv X., He C., Hua G., Tsai M.-Y., Davis J.S. (2013). The G-protein-coupled estrogen receptor agonist G-1 suppresses proliferation of ovarian cancer cells by blocking tubulin polymerization. Cell Death Dis..

[125] Liu H., Yan Y., Wen H., Jiang X., Cao X., Zhang G., Liu G. (2014). A novel estrogen receptor GPER mediates proliferation induced by 17β-estradiol and selective GPER agonist G-1 in estrogen receptor α (ERα)-negative ovarian cancer cells. Cell Biol. Int..

[126] Albanito L., Madeo A., Lappano R., Vivacqua A., Rago V., Carpino A., Oprea T.I., Prossnitz E.R., Musti A.M., Andò S. (2007). G Protein–Coupled Receptor 30 (GPR30) Mediates Gene Expression Changes and Growth Response to 17β-Estradiol and Selective GPR30 Ligand G-1 in Ovarian Cancer Cells. Cancer Res..

[127] Scaling A.L., Prossnitz E.R., Hathaway H.J. (2014). GPER Mediates Estrogen-Induced Signaling and Proliferation in Human Breast Epithelial Cells and Normal and Malignant Breast. Horm. Cancer.

[128] Filardo E.J., Quinn J.A., Bland K.I., Frackelton A.R. (2000). Estrogen-Induced Activation of Erk-1 and Erk-2 Requires the G Protein-Coupled Receptor Homolog, GPR30, and Occurs via Trans-Activation of the Epidermal Growth Factor Receptor through Release of HB-EGF. Mol. Endocrinol..

[129] Zhao Y., Zhao M.-F., Yang M.-L., Wu T.-Y., Xu C.-J., Wang J.-M., Li C.-J., Li X. (2020). G Protein–Coupled Receptor 30 Mediates the Anticancer Effects Induced by Eicosapentaenoic Acid in Ovarian Cancer Cells. Cancer Res. Treat..

[130] Sun Q., Gong T., Liu M., Ren S., Yang H., Zeng S., Zhao H., Chen L., Ming T., Meng X. (2022). Shikonin, a naphthalene ingredient: Therapeutic actions, pharmacokinetics, toxicology, clinical trials and pharmaceutical researches. Phytomedicine.

[131] Liu X., Yang Y., Tang X., Guo L., Tang X., Zhu T., Zhao T., Zhang W., Zhang P. (2022). Shikonin Mediates Apoptosis through G Protein-Coupled Estrogen Receptor of Ovarian Cancer Cells. Evid.-Based Complement. Altern. Med..

[132] Girgert R., Emons G., Gründker C. (2018). Estrogen Signaling in ERα-Negative Breast Cancer: ERβ and GPER. Front. Endocrinol..

[133] Han N., Heublein S., Jeschke U., Kuhn C., Hester A., Czogalla B., Mahner S., Rottmann M., Mayr D., Schmoeckel E. (2021). The G-Protein-Coupled Estrogen Receptor (GPER) Regulates Trimethylation of Histone H3 at Lysine 4 and Represses Migration and Proliferation of Ovarian Cancer Cells In Vitro. Cells.

[134] Skrzypczak M., Schüler S., Lattrich C., Ignatov A., Ortmann O., Treeck O. (2013). G protein-coupled estrogen receptor (GPER) expression in endometrial adenocarcinoma and effect of agonist G-1 on growth of endometrial adenocarcinoma cell lines. Steroids.

[135] Krakstad C., Trovik J., Wik E., Engelsen I.B., Werner H.M.J., Birkeland E., Raeder M.B., Øyan A.M., Stefansson I.M., Kalland K.H. (2012). Loss of GPER identifies new targets for therapy among a subgroup of ERα-positive endometrial cancer patients with poor outcome. Br. J. Cancer.

[136] Zhang L., Li Y., Lan L., Liu R., Wu Y., Qu Q., Wen K. (2016). Tamoxifen has a proliferative effect in endometrial carcinoma mediated via the GPER/EGFR/ERK/cyclin D1 pathway: A retrospective study and an in vitro study. Mol. Cell Endocrinol..

[137] Lv Q.-Y., Xie B.-Y., Yang B.-Y., Ning C.-C., Shan W.-W., Gu C., Luo X.-Z., Chen X.-J., Zhang Z.-B., Feng Y.-J. (2017). Increased TET1 Expression in Inflammatory Microenvironment of Hyperinsulinemia Enhances the Response of Endometrial Cancer to Estrogen by Epigenetic Modulation of GPER. J. Cancer.

[138] Du G.-Q., Zhou L., Chen X.-Y., Wan X.-P., He Y.-Y. (2012). The G protein-coupled receptor GPR30 mediates the proliferative and invasive effects induced by hydroxytamoxifen in endometrial cancer cells. Biochem. Biophys. Res. Commun..

[139] De Marco P., Bartella V., Vivacqua A., Lappano R., Santolla M.F., Morcavallo A., Pezzi V., Belfiore A., Maggiolini M. (2013). Insulin-like growth factor-I regulates GPER expression and function in cancer cells. Oncogene.

[140] Wan J., Yin Y., Zhao M., Shen F., Chen M., Chen Q. (2017). The positivity of G-protein-coupled receptor-30 (GPR 30), an alternative estrogen receptor is not different between type 1 and type 2 endometrial cancer. Oncotarget.

[141] He Y.-Y., Du G.-Q., Cai B., Yan Q., Zhou L., Chen X.-Y., Lu W., Yang Y.-X., Wan X.-P. (2012). Estrogenic transmembrane receptor of GPR30 mediates invasion and carcinogenesis by endometrial cancer cell line RL95-2. J. Cancer Res. Clin. Oncol..

[142] Ge X., Guo R., Qiao Y., Zhang Y., Lei J., Wang X., Li L., Hu D. (2013). The G Protein–Coupled Receptor GPR30 Mediates the Nontranscriptional Effect of Estrogen on the Activation of PI3K/Akt Pathway in Endometrial Cancer Cells. Int. J. Gynecol. Cancer.

[143] Sun P., Sehouli J., Denkert C., Mustea A., Könsgen D., Koch I., Wei L., Lichtenegger W. (2005). Expression of estrogen receptor-related receptors, a subfamily of orphan nuclear receptors, as new tumor biomarkers in ovarian cancer cells. J. Mol. Med..

[144] Fujimoto J., Alam S.M., Jahan I., Sato E., Sakaguchi H., Tamaya T. (2007). Clinical implication of estrogen-related receptor (ERR) expression in ovarian cancers. J. Steroid Biochem. Mol. Biol..

[145] Schüler-Toprak S., Weber F., Skrzypczak M., Ortmann O., Treeck O. (2021). Expression of estrogen-related receptors in ovarian cancer and impact on survival. J. Cancer Res. Clin. Oncol..

[146] Huang W., Chen L., Sun P. (2022). ERRα expression in ovarian cancer and promotes ovarian cancer cells migration in vitro. Arch. Gynecol. Obstet..

[147] Ghilardi C., Moreira-Barbosa C., Brunelli L., Ostano P., Panini N., Lupi M., Anastasia A., Fiordaliso F., Salio M., Formenti L. (2022). PGC1α/β Expression Predicts Therapeutic Response to Oxidative Phosphorylation Inhibition in Ovarian Cancer. Cancer Res..

[148] Luo C., Widlund H.R., Puigserver P. (2016). PGC-1 Coactivators: Shepherding the Mitochondrial Biogenesis of Tumors. Trends Cancer.

[149] LeBleu V.S., O’Connell J.T., Gonzalez Herrera K.N.G., Wikman H., Pantel K., Haigis M.C., De Carvalho F.M., Damascena A., Domingos Chinen L.T., Rocha R.M. (2014). PGC-1α mediates mitochondrial biogenesis and oxidative phosphorylation in cancer cells to promote metastasis. Nat. Cell Biol..

[150] Huang X., Ruan G., Liu G., Gao Y., Sun P. (2020). Immunohistochemical Analysis of PGC-1α and ERRα Expression Reveals Their Clinical Significance in Human Ovarian Cancer. OncoTargets Ther..

[151] Huang X., Ruan G., Sun P. (2021). Estrogen-related receptor alpha copy number variation is associated with ovarian cancer histological grade. J. Obstet. Gynaecol. Res..

[152] Lam S.S., Mak A.S., Yam J.W., Cheung A.N., Ngan H.Y., Wong A.S. (2014). Targeting Estrogen-Related Receptor Alpha Inhibits Epithelial-to-Mesenchymal Transition and Stem Cell Properties of Ovarian Cancer Cells. Mol. Ther..

[153] Krishna B.M., Chaudhary S., Mishra D.R., Naik S.K., Suklabaidya S., Adhya A.K., Mishra S.K. (2018). Estrogen receptor α dependent regulation of estrogen related receptor β and its role in cell cycle in breast cancer. BMC Cancer.

[154] Yu S., Wong Y.C., Wang X.H., Ling M.T., Ng C.F., Chen S., Chan F.L. (2008). Orphan nuclear receptor estrogen-related receptor-β suppresses in vitro and in vivo growth of prostate cancer cells via p21WAF1/CIP1 induction and as a potential therapeutic target in prostate cancer. Oncogene.

[155] Watanabe A., Kinoshita Y., Hosokawa K., Mori T., Yamaguchi T., Honjo H. (2006). Function of Estrogen-Related Receptor α in Human Endometrial Cancer. J. Clin. Endocrinol. Metab..

[156] Sun P., Mao X., Gao M., Huang M., Chen L., Ruan G., Huang W., Braicu E.I., Sehouli J. (2018). Novel endocrine therapeutic strategy in endometrial carcinoma targeting estrogen-related receptor α by XCT790 and siRNA. Cancer Manag. Res..

[157] Matsushima H., Mori T., Ito F., Yamamoto T., Akiyama M., Kokabu T., Yoriki K., Umemura S., Akashi K., Kitawaki J. (2016). Anti-tumor effect of estrogen-related receptor alpha knockdown on uterine endometrial cancer. Oncotarget.

[158] Fujimoto J., Sato E. (2009). Clinical implication of estrogen-related receptor (ERR) expression in uterine endometrial cancers. J. Steroid Biochem. Mol. Biol..

[159] Huang M., Chen L., Mao X., Liu G., Gao Y., You X., Gao M., Sehouli J., Sun P. (2020). ERRα inhibitor acts as a potential agonist of PPARγ to induce cell apoptosis and inhibit cell proliferation in endometrial cancer. Aging.

[160] Mao X., Lei H., Yi T., Su P., Tang S., Tong Y., Dong B., Ruan G., Mustea A., Sehouli J. (2022). Lipid reprogramming induced by the TFEB-ERRα axis enhanced membrane fluidity to promote EC progression. J. Exp. Clin. Cancer Res..

[161] Deblois G., St-Pierre J., Giguère V. (2013). The PGC-1/ERR signaling axis in cancer. Oncogene.

[162] De Vitto H., Ryu J., Calderon-Aparicio A., Monts J., Dey R., Chakraborty A., Lee M.-H., Bode A.M., Dong Z. (2020). Estrogen-related receptor alpha directly binds to p53 and cooperatively controls colon cancer growth through the regulation of mitochondrial biogenesis and function. Cancer Metab..

[163] Chen L., Mao X., Huang M., Lei H., Xue L., Sun P. (2020). PGC-1α and ERRα in patients with endometrial cancer: A translational study for predicting myometrial invasion. Aging.

[164] Yoriki K., Mori T., Kokabu T., Matsushima H., Umemura S., Tarumi Y., Kitawaki J. (2019). Estrogen-related receptor alpha induces epithelial-mesenchymal transition through cancer-stromal interactions in endometrial cancer. Sci. Rep..

[165] Huang X., Wang X., Shang J., Zhaang Z., Cui B., Lin Y., Yang Y., Song Y., Yu S., Xia J. (2018). Estrogen related receptor alpha triggers the migration and invasion of endometrial cancer cells via up regulation of TGFB1. Cell Adhes. Migr..

[166] Kokabu T., Mori T., Matsushima H., Yoriki K., Kataoka H., Tarumi Y., Kitawaki J. (2019). Antitumor effect of XCT790, an ERRα inverse agonist, on ERα-negative endometrial cancer cells. Cell Oncol..

[167] Sun P.-M., Gao M., Wei L.-H., Mustea A., Wang J.-L., Könsgen D., Lichtenegger W., Sehouli J. (2006). An estrogen receptor alpha-dependent regulation of estrogen receptor-related receptor alpha in the proliferation of endometrial carcinoma cells. Int. J. Gynecol. Cancer.

[168] Tong Y., Huang M., Chen L., Lei H., Lin H., Mao X., Sun P. (2022). ERRγ, a Novel Biomarker, Associates with Pathoglycemia of Endometrial Cancer to Predict Myometrial Invasion. J. Oncol..

[169] Yamamoto T., Mori T., Sawada M., Kuroboshi H., Tatsumi H., Yoshioka T., Matsushima H., Iwasaku K., Kitawaki J. (2012). Estrogen-Related Receptor-γ Regulates Estrogen Receptor-α Responsiveness in Uterine Endometrial Cancer. Int. J. Gynecol. Cancer.

[170] Byrne F.L., Martin A.R., Kosasih M., Caruana B.T., Farrell R. (2020). The Role of Hyperglycemia in Endometrial Cancer Pathogenesis. Cancers.

[171] Ko E.M., Walter P., Clark L., Jackson A., Franasiak J., Bolac C., Havrilesky L., Secord A.A., Moore D.T., Gehrig P.A. (2014). The complex triad of obesity, diabetes and race in Type I and II endometrial cancers: Prevalence and prognostic significance. Gynecol. Oncol..

[172] Onstad M.A., Schmandt R.E., Lu K.H. (2016). Addressing the Role of Obesity in Endometrial Cancer Risk, Prevention, and Treatment. J. Clin. Oncol..

[173] Yoshihara E., Wei Z., Lin C.S., Fang S., Ahmadian M., Kida Y., Tseng T., Dai Y., Yu R.T., Liddle C. (2016). ERRγ Is Required for the Metabolic Maturation of Therapeutically Functional Glucose-Responsive β Cells. Cell Metab..

[174] Misra J., Kim D.-K., Choi H.-S. (2017). ERRγ: A Junior Orphan with a Senior Role in Metabolism. Trends Endocrinol. Metab..

[175] Soundararajan A., Prabu P., Mohan V., Gibert Y., Balasubramanyam M. (2019). Novel insights of elevated systemic levels of bisphenol-A (BPA) linked to poor glycemic control, accelerated cellular senescence and insulin resistance in patients with type 2 diabetes. Mol. Cell Biochem..

